# Using IRTree Models to Promote Selection Validity in the Presence of Extreme Response Styles

**DOI:** 10.3390/jintelligence11110216

**Published:** 2023-11-17

**Authors:** Victoria L. Quirk, Justin L. Kern

**Affiliations:** Department of Educational Psychology, University of Illinois at Urbana-Champaign, Champaign, IL 61820, USA; vquirk3@illinois.edu

**Keywords:** item response theory, item response trees, IRTree models, response process, non-content variability, extreme response styles, Likert scales, selection validity, classification accuracy, adverse impact

## Abstract

The measurement of psychological constructs is frequently based on self-report tests, which often have Likert-type items rated from “Strongly Disagree” to “Strongly Agree”. Recently, a family of item response theory (IRT) models called IRTree models have emerged that can parse out content traits (e.g., personality traits) from noise traits (e.g., response styles). In this study, we compare the selection validity and adverse impact consequences of noise traits on selection when scores are estimated using a generalized partial credit model (GPCM) or an IRTree model. First, we present a simulation which demonstrates that when noise traits do exist, the selection decisions made based on the IRTree model estimated scores have higher accuracy rates and have less instances of adverse impact based on extreme response style group membership when compared to the GPCM. Both models performed similarly when there was no influence of noise traits on the responses. Second, we present an application using data collected from the Open-Source Psychometrics Project Fisher Temperament Inventory dataset. We found that the IRTree model had a better fit, but a high agreement rate between the model decisions resulted in virtually identical impact ratios between the models. We offer considerations for applications of the IRTree model and future directions for research.

## 1. Introduction

Personality tests have become as common in job applications as attaching a resume. And for good reason: many personality traits demonstrate predictive validity for job outcomes, like organizational commitment (Farrukh et al. 2017), workplace safety behaviors (Beaus et al. 2015), and job performance (Barrick and Mount 1991). The use of personality testing in selection procedures has also been associated with higher levels of minority representation in organizations (Ng and Sears 2010). Personality is often measured through responses to Likert-type items (Likert 1932). These items are typically formatted as a statement, like “I am usually on time”, followed by a set of response categories in varying degrees of “Agree” and “Disagree”, with, potentially, a “Neutral” option.

One potential source of noise in Likert-scale tests is response styles. Response styles can be thought of as a tendency to systematically select responses as a function of item format rather than item content, which can decrease the validity of a test (Cronbach 1946, 1950). Likert-type items are particularly prone to response style effects (response style effects, if unaccounted for, can negatively impact model fit and estimate accuracy through the false attribution of responses to content-related traits). There is a large body of recent research that demonstrates the efficacy of various modeling techniques in the presence of different types of response styles, such as midpoint response styles (Böckenholt 2017; Baumgartner and Steenkamp 2001), acquiescence responses styles (Park and Wu 2019; Baumgartner and Steenkamp 2001), omission response styles (Jeon and De Boeck 2016), and extreme response styles (Böckenholt 2017; Park and Wu 2019; Baumgartner and Steenkamp 2001). In the current study, we focus on extreme response styles and their impact on selection validity and adverse impact in classification decisions, though our approach could be modified for any theorized sequential response process. An extreme response style is related to the tendency of a person to select a “Strong” response category, regardless of whether they initially choose to agree or disagree with an item. In recent years, several IRT models that can account for response styles have been proposed; these models are successful at identifying response styles when they occur (Böckenholt 2017; Böckenholt and Meiser 2017; De Boeck and Partchev 2012; Jeon and De Boeck 2016; Leventhal 2019; Park and Wu 2019; Huang 2016). However, there is little research on how response styles could impact selection decisions when present, and how selection decisions can be aided by accounting for response styles using these models. 

Regarding extreme response styles, it is possible that responses may also vary in extremity depending on the direction of agreement. When this occurs, this violates the assumption of directional invariance—the assumption that the extreme response tendency is not dependent upon the level of agreement. Directional non-invariance can occur on either the person level, the item level, or both (Jeon and De Boeck 2019). On the person level, participants may have a different level of extreme response style trait based on whether they initially agree or disagree with the item. For example, a participant seeking a job may believe that strongly disagreeing with an item would reduce their appeal to an employer but that strongly agreeing with an item would be more acceptable. On the item level, some items may have higher thresholds for a strong negative response. For example, an item regarding a controversial topic may be more difficult to strongly agree with than to strongly disagree with. In the current study, we compare model robustness to situations of directional invariance occurring at the item level, though different constraints could be imposed to align with other theories of potential invariance. While the previous research demonstrates that relaxing the assumption of invariance using IRTree models improves the model fit in many cases (Jeon and De Boeck 2019), there is a gap in the literature regarding how directional non-invariance on the item level may impact classification validity under various model structures that do not specifically account for this type of non-invariance. When implementing any kind of test as a method for candidate screening, it is important to consider adverse impact. The Equal Employment Opportunity Commission defines adverse impact as “a substantially different rate of selection in hiring, promotion, or other employment decision” which places members of a protected group at a disadvantage (Equal Employment Opportunity Commission 1979). There is a simple four-step process used to determine whether adverse impact has taken place. First, calculate the selection rate for each group. Second, observe which group has the highest selection rate. Third, calculate the impact ratio by dividing the selection rate for each group by the highest selection rate. Fourth, compare the impact ratio to a predetermined cutoff. Typically, an impact ratio of less than 0.80 or greater than 1.25 is considered to be evidence of adverse impact (Uniform Guidelines on Employee Selection Procedures 1978).

For example, if 10 out of 50 women who applied to a job were hired and 38 out of 150 men were hired, the impact ratio for women would be:

(1) $$Impact\;Ratio=\frac{Reference\;Selection\;Rate}{Focal\;Selection\;Rate}=\frac{\frac{10}{50}}{\frac{38}{150}}=0.79,$$

The impact ratio of 0.79 indicates that the hiring rate of women is 79% of the hiring rate of men, which would be considered evidence of adverse impact in hiring practices. Adverse impact in selection practices constitutes illegal discrimination (Civil Rights Act 1964) and has many negative consequences for an organization, including reduced diversity (Ng and Sears 2010). Previous research has found group differences in the level of extreme response style that occur on the basis of gender (De Jong et al. 2008), race (Bachman and O’Malley 1984), and age (Batchelor and Miao 2016). If unaccounted for, extreme response styles can indirectly introduce bias into the way a test is scored (Leventhal 2019). In this article, we examine how structuring a model to align with a theorized response process has the potential to increase validity, moderate the relationship between demographics and classification decisions, and reduce adverse impact through accounting for noise traits.

The rest of this article is as follows. First, we describe our models for comparison: the generalized partial credit model and the item response tree (IRTree) model. Second, a simulation study examining selection validity and impact ratios in the presence of extreme response styles is described, including a comparison of these between the two models. Third, we apply these models to a real dataset to compare model fit, classification decisions, and impact ratios. Fourth, the article concludes with a brief discussion and suggestions for future research.

## 2. Models

In this section, we briefly describe each of the models used in the data generation and the fitting of the data in both the simulation and the applied example. 

### 2.1. IRTree Model

**Figure 1:**
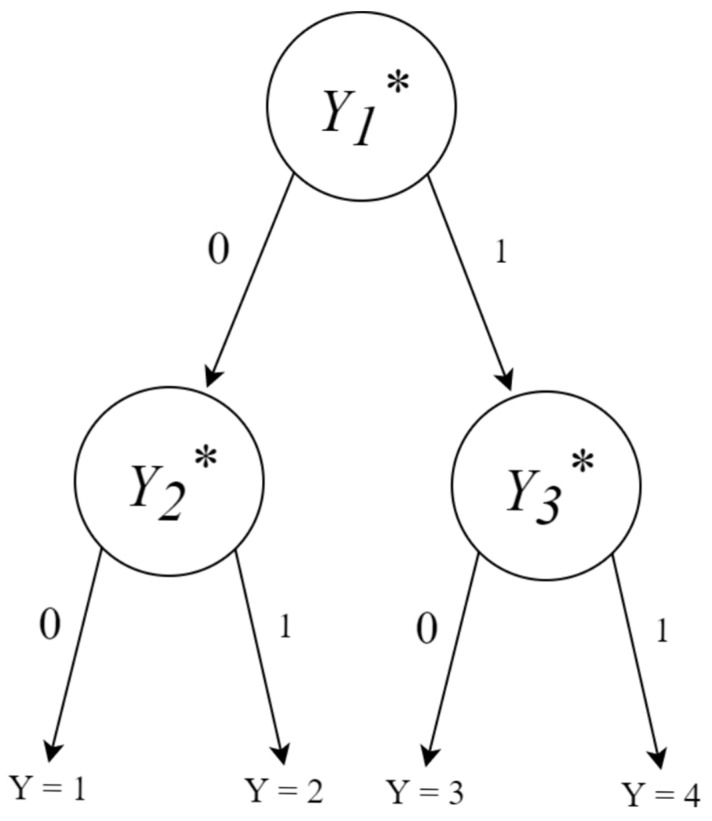
One of the possible structures for an IRTree model, where each node represents a different IRT model and 0 s and 1 s represent decisions made at each node. The asterisk notation ($Y_{k}^{*}$) denotes pseudo-item responses at each branch resulting in the observed response Y.

IRTree models refer to the recently developed family of models that utilize an IRT model at each node of a theorized response process (Böckenholt 2012; De Boeck and Partchev 2012; Jeon and De Boeck 2016). There are many ways that an IRTree model can be structured in accordance with the underlying theorized response process. These processes can be visualized as a decision tree, like the one given in Figure 1. For alternative tree structures, see Böckenholt (2012), De Boeck and Partchev (2012), Böckenholt (2017), and Meiser et al. (2019). In Figure 1, the Likert scale responses $Y_{i}=1,\ldots ,\;4$ are shown to be the end nodes, or leaves, of the decision tree. At the first node, denoted by $Y_{1}^{*}$, a person decides whether to agree (1) or disagree (0) with the statement based on their content trait level. At the second level of nodes, $Y_{2}^{*}$ or $Y_{3}^{*}$, depending on whether the person decided to disagree or agree with the statement, respectively, the person decides whether to respond strongly (1) or moderately (0) based on their level of extreme response style and their level of content trait. The structural flexibility of the IRTree approach allows the estimation of unique parameters based on each node that is a part of the response process. This enables the parsing out of the content trait from any non-content, or nuisance, traits. It also allows the first-level node decision to impact the probabilities of the second-level node decisions. In the extreme response style literature, when the first-level node decision does not impact the second-level node probabilities, it is known as directional invariance (i.e., the probability of an extreme response is invariant relative to the direction of agreement; Jeon and De Boeck 2019). 

The decision tree shown in Figure 1 is used in the current study. Specifically, the first level node decision is assumed to be controlled by a content-specific trait $\theta_{C}$, and the second level node decisions are assumed to be controlled by both a common extreme response style trait $\theta_{E}$ and the content-specific trait, $\theta_{C}$. This may be likely in real datasets, where a person that has a very high level of content trait might be more likely to “Strongly Agree” with an item, even if they have only a moderate or low level of extreme response style trait. Due to this model being essentially a bifactor model, for identification purposes, $\Sigma_{\theta }=I_{2}$.

The probabilities for each node response are given by a two-parameter logistic (2PL) model. For node 1:

(2) $$P\left(Y_{ij1}^{*}=1|\theta_{jC}\right)={\left[1+\exp\left(-\left(\alpha_{i1C}\theta_{jC}+\beta_{i1}\right)\right)\right]}^{-1},$$

where $Y_{ij1}^{*}$ represents the agree–disagree pseudo-response for person j on item i, and $\alpha_{i1C}$ and $\beta_{i1}$ represent the pseudo-item slope and intercept parameters for item i at node 1, respectively. For nodes 2 and 3, we have implemented a similar model to that described in Meiser et al. (2019) to demonstrate a multidimensional decision.

(3) $$P\left(Y_{ijk}^{*}=1|\theta_{jC},\theta_{jE}\right)={\left[1+\exp\left(-\left(\alpha_{ikC}\theta_{jC}+\alpha_{ikE}\theta_{jE}+\beta_{ik}\right)\right)\right]}^{-1},$$

where $Y_{ijk}^{*}$ represents the strength of agreement (either 0: moderately or 1: strongly) pseudo-item response for person j on item i node k; $\alpha_{ikC}$ and $\alpha_{ikE}$ represent slope parameters for the content and extreme response style traits, respectively, on item *i* at node k; and $\beta_{ik}$ represents the intercept parameter for item i at node k. This model represents a situation in which the response styles are present in the data and both the content trait and the extreme response style trait have an impact on the decision to strongly or moderately endorse a directional response.

### 2.2. Generalized Partial Credit Model (GPCM)

The GPCM is a polytomous extension of the 2PL model that can be used for ordinal data (Muraki 1992). It is reasonable to use this model to estimate the latent traits of polytomous Likert-type items; so, we use this as our standard for model comparison. A unidimensional model was chosen to represent situations in which there is little known about the relationship between the content trait and the response style trait. Under this model, the probability $P_{ix}(\theta_{j})$ for selecting category x on item i for person j is:

(4) $$P\left(X_{i}=x|\theta_{j}\right)=P_{ix}\left(\theta_{j}\right)=\frac{\exp\left(\sum_{j=0}^{x}\alpha_{i}\left(\theta_{j}-\delta_{ij}\right)\right)}{\sum_{r=0}^{m_{i}}\exp\left(\sum_{k=0}^{r}\alpha_{i}\left(\theta_{j}-\delta_{ik}\right)\right)},\;x\in \{0,\;\ldots ,\;m_{i}\},$$

where $m_{i}+1$ is the number of response categories, $\delta_{ix}$ is the step difficulty associated with response x on item i, $\alpha_{i}$ is the discrimination parameter for item i, and $\alpha_{i}\left(\theta_{j}-\delta_{i0}\right)\;\equiv 0$. Here, the value of $\delta_{ix}$ indicates the point on the latent trait scale where the probability of a response in category x is equal to the probability of a response in category x−1 on the item. No extreme response style trait is estimated in this model.

We can think of the two data-generating models (the GPCM and the IRTree) as two extremes: the GPCM, assuming no response style or that the response style is not differentiable from the content trait for any person, and the IRTree, assuming the decision to strongly endorse a response is dependent on both a content trait and some extreme response style trait rather than content trait alone. It is worth noting that there are many possible in-between states that may be true in practice, such as when there are thought to be multiple groups for the response style trait. We recommend the following readings for alternative methods of implementing multidimensional nodes (Khorramdel and von Davier 2014; Khorramdel et al. 2019) or mixture modeling for multiple groups (Tijmstra et al. 2018; Khorramdel et al. 2019; Kim and Bolt 2021), respectively.

To investigate the implications of unaccounted for non-invariance, we also tested our described IRTree model (which does not specifically account for directional non-invariance but estimates all pseudo-item parameters separately for each node) and the GPCM (which assumes directional invariance holds) on the data generated using an IRTree model that demonstrates directional non-invariance. By not specifying either of the data-fitting models (the IRTree or the GPCM) used to account for directional non-invariance, we can test the robustness of both models to this confounder in situations where there is little knowledge about the directional invariance properties of the scale. In the context of this study, directional non-invariance on the item level can be interpreted as a uniformly lower threshold to “Strongly” endorse a response if the first level decision was “Agree”. 

## 3. Simulation Study

### 3.1. Methods

The primary interest of this study is in whether there is a decrease in selection accuracy when the models are misspecified. A further question is whether the level of decreasing selection accuracy is dependent on the fitted model. In doing so, this may shed light on whether one model is a “safer bet” than the other in situations where little may be known about the response process. We also wish to examine the practical implications of differences in selection due to model selection, such as whether the examinees with high or low response style levels are more or less likely to be selected under one model condition than the other. 

To investigate the selection validity between the models, a simulation study is conducted. In this study, the simulated responses were first generated from a data-generating model. For each set of responses, both the IRTree model and the GPCM were then fitted to the data using the R package *mirt* (Chalmers 2012). Finally, a proportion of the examinees were selected according to the set selection rate. Given the selection rate, a determination can be made as to which examinees should have been selected according to their true content trait values and whether they were correctly selected. It was expected that when the fitted model matched the form of the data-generating model, that the selection accuracy would be maximized. 

In this study, four factors were varied. First, there were three levels of sample size (N=500, 1000, or 2000). Second, there were two levels of test length (n=25 or 50). Third, there were three levels of data-generating model: GPCM; an IRTree model with item-level directional invariance ($\beta_{i3}=\beta_{i2}+\delta$, where δ=0.0); and an IRTree model without item-level directional invariance ($\beta_{i3}=\beta_{i2}+\delta$, where δ=0.6). The directional non-invariance condition means that the probability of a “strong” response is larger for those who agree than those who disagree. This could occur when items display a social desirability effect. Because this is similar to the concept of uniform differential item functioning (DIF), we selected a δ value of 0.0 to signify directional invariance and a value of 0.6 to signify directional non-invariance in accordance with the DIF simulation literature. Item difficulty parameter shifts of 0.5 or greater are typically considered to be medium to large differences in simulations of DIF for DIF detection studies (Battauz 2019; Finch 2022). Lastly, there were nine levels of selection cut scores (10%, 15%, 20%, 25%, 50%, 60%, 70%, 80%, and 90%). These levels were selected in accordance with prior research in pre-employment screening using personality tests (Mueller-Hanson et al. 2003). All the factors were fully crossed, for a total of 3×3×2×9=162  conditions, with each condition replicated 100 times. 

For each replicate, one set of person and item parameters was generated, with the item parameters generated according to the level of the generating model factor: either the GPCM, the IRTree with item-level directional invariance, or the IRTree model with item-level directional non-invariance. The latent trait levels for each person for each generating model were drawn from a two-dimensional multivariate normal distribution with $\mu_{\theta }=0_{2}$, $\Sigma_{\theta }=I_{2}$. 

The slope parameters for both the GPCM and node 1 of the IRTree model were generated from Unif(1.0, 2.5). For the IRTree model, this represents the unidimensional pseudo-item slope for the content trait of the first-level decision. At the second level, we assume that the extreme response style is more strongly related to the second-level decision than the content trait. As such, we also generate the slope parameter $a_{i2E}$ from $Unif\left(1.0,\;2.5\right),$ but we generate the slope parameter $a_{i2C}$ from Unif(0.5, 1.0). For the IRTree models, the magnitude of the pseudo-item slopes at the second and third nodes are equal. In this way, when there is no directional invariance, the probabilities between nodes 2 and 3 are shifted uniformly relative to each other. However, the pseudo-item slopes associated with the content trait are reversed for node 3, $a_{i2C}=-a_{i3C}$. This is performed so that the higher values of $\theta_{C}$ are always associated with a higher probability of selecting a higher agreement response option. This reversal is not needed for the slopes associated with $\theta_{E}$, as the decision to give an extreme response is indicated with a pseudo-item response of 1 at both nodes. As this is the case, higher values of $\theta_{E}$ are associated with a higher probability of selecting a “Strongly –” option over a moderate response category. 

The three threshold parameters of $\delta_{ix}$, x∈{1, 2, 3}, for the GPCM-generated items were generated from normal distributions with means of μ, where μ ∈ {1, 0,−1} and the standard deviations of σ = 1. The intercept parameters for nodes 1 and 2 of the IRTree models were generated from a truncated standard normal distribution, truncated at ±1. The intercept parameter for node 3 was calculated for each IRTree model (i.e., $\beta_{i3}=\beta_{i2}+\delta$ where δ=0.0 for the IRTree model with directional invariance or 0.6 for the model with directional non-invariance). The response data were then simulated for all three models. 

To fit the IRTree model to the simulated data, the responses from each model were further broken down into pseudo-responses to each pseudo-item, using the response mapping convention shown in Table 1. When fitting the IRTree models, no item parameter constraints were used; thus, each node had separately estimated slope and intercept parameters. Furthermore, for identification purposes, the traits were estimated to have zero correlation. After fitting each model, the latent traits were estimated using a maximum a posteriori (MAP) estimator. The selection decisions were made based on the content trait estimate of each model and the selection rate for that replicate. Each person’s level of extreme response style was estimated using the IRTree model and labeled as either low (bottom third), moderate (middle third), or high (top third). 

Correct decision rate, sensitivity, specificity, positive predictive value, and negative predictive value were calculated for each model and selection rate combination across all levels of extreme response style. The observed selection rate, correct decision rate, sensitivity, specificity, positive predictive value, negative predictive value, and impact ratio were then evaluated by level of extreme response style (low, moderate, or high). The observed selection rate was calculated by dividing the number of examinees selected by the total number of examinees. When the decision that should have been made based on a true score was the same as the observed decision, a correct decision was made. The correct decision rate is the number of correct decisions divided by sample size. Sensitivity is the number of correctly selected examinees divided by the number of examinees that should have been selected. Specificity is the number of correctly not-selected examinees divided by the number of examinees that should not have been selected. The positive predictive value is the number of correctly selected examinees divided by the total number of examinees selected. Negative predictive value is the number of correctly not-selected examinees divided by the total number of examinees not selected. Impact ratio was calculated by dividing the observed selection rate of the focal group (low or high extreme response style) by the observed selection rate of the reference group (moderate extreme response style). In this simulation, we consider impact ratios greater than 1.25 or less than 0.80 to be evidence of adverse impact. This allows us to detect adverse impact regardless of whether the focal or reference group has a higher selection rate. 

To compare the models fitted to the same generated data, both the AIC and the BIC model fit statistics were used for each model combination. To further confirm this method of model selection for the applied example, we found that in 100% of the cases in the simulation both of the model fit indices were the lowest when the model was correctly specified. In other words, when there was no evidence of response styles, the GPCM showed a better fit than the IRTree model, and when there was evidence of response styles, the IRTree model showed a better fit than the GPCM. 

### 3.2. Results

**Figure 2:**
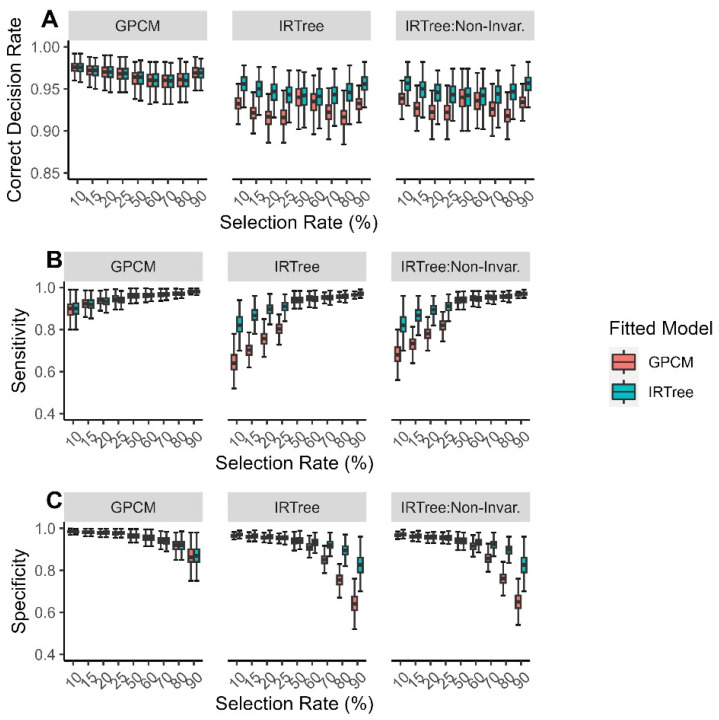
Simulation findings for each generating model across all levels of extreme response style for (**A**) correct decision rate; (**B**) sensitivity; (**C**) specificity, with color representing the fitted model.

**Figure 3:**
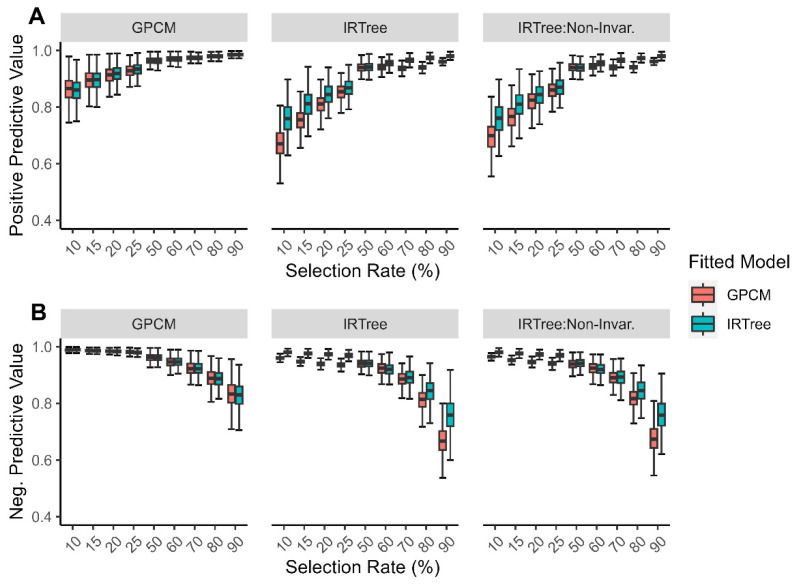
Simulation findings for each generating model across all levels of extreme response style for (**A**) positive predictive value; (**B**) negative predictive value, with color representing the fitted model.

The findings for each outcome variable were very consistent across the sample sizes and test lengths, though increases in each resulted in slight increases in power. In particular, it was found that selection rate was highly influential for the outcomes. The mean and standard deviations of each outcome according to each condition are reported in the Supplementary Materials (Tables S1–S6); see also Figure 2 and Figure 3. The findings by the data-generating model, fitted model, and selection rate are described in more detail below.

#### 3.2.1. Overall Findings

When the data are generated with a GPCM, both of the fitted models perform similarly well, with the GPCM generally having greater correct decision rates than the IRTree model. However, when the data are generated with an IRTree model (with or without directional invariance), we find that the IRTree model demonstrates a much higher level of correct decision rates and fluctuates very little across the selection rate, while the GPCM demonstrates much lower levels of correct decision rates and does vary across the selection rate. When the data are generated with an IRTree model, the GPCM demonstrates the highest correct decision rates in the mid-range selection rate conditions and much lower correct decision rates when the selection rates are very high or very low. In compiling the findings for each outcome variable, it becomes clear that these high correct decision rates are a result of the sensitivity and specificity both being high in this range, while the sensitivity tends to be low when the selection rate is low, and the specificity tends to be low when the selection rate is high. While the IRTree model is not as susceptible to these effects, it is important to note that the mid-range selection rates may yield higher correct decision rates regardless of the model specification.

In general, the sensitivity increased as the selection rate increased. When the data were generated with a GPCM, both fitted models performed almost equally well, with the GPCM generally having greater sensitivity than the IRTree model. When the data were generated with an IRTree model and fitted with a GPCM, the sensitivity varied greatly depending on the generating model and the selection rate. The sensitivity when the data were generated with an IRTree model and fitted with an IRTree model was much higher and more consistent across the generating model and selection rate than when the data were fitted with a GPCM.

In general, the specificity decreased as the selection rate increased. When the data were generated with a GPCM, both of the fitted models performed almost equally well, with the GPCM generally having greater specificity than the IRTree model. When the data were generated with an IRTree, there was much greater difference in the performance of each model, with the IRTree fitted data demonstrating much higher specificity and more consistent specificity across the generating model and selection rate than when the data were fitted with a GPCM. The overall findings for the correct decision rate, sensitivity, and specificity can be found in Figure 2.

The positive predictive values increased as the selection rate increased. When the data were generated with a GPCM, the models displayed nearly identical positive predictive values, but when the data were generated with an IRTree, the IRTree fitted model tended to have higher positive predictive values than the fitted GPCM. 

The negative predictive values decreased as the selection rate increased. When the data were generated with a GPCM, the models displayed nearly identical negative predictive values, but when the data were generated with an IRTree, the IRTree fitted model tended to have higher negative predictive values than the fitted GPCM. The overall findings for the positive and negative predictive value can be found in Figure 3. 

Regardless of the generating model, the IRTree model displays very similar correct decision rates, sensitivity, specificity, positive predictive values, and negative predictive values across the selection rate levels, suggesting that the IRTree model is less susceptible to reduced selection validity in cases of model misspecification. In other words, when an IRTree model is fitted to the data, regardless of whether or not there are extreme response styles in the data, the selection validity is high, though it is slightly improved when there is evidence of extreme response styles in the data. On the other hand, the GPCM tends to have high selection validity when there are not extreme response styles in the data, but the performance tends to be reduced when there are extreme response styles in the data. The degree of this reduction in model performance depends on the level of the extreme response style. The findings are also very similar across the fitted model performances when the data are generated with directional invariance or directional non-invariance, implying that both models are robust to this confounder. 

#### 3.2.2. By Level of Extreme Response Style

**Figure 4:**
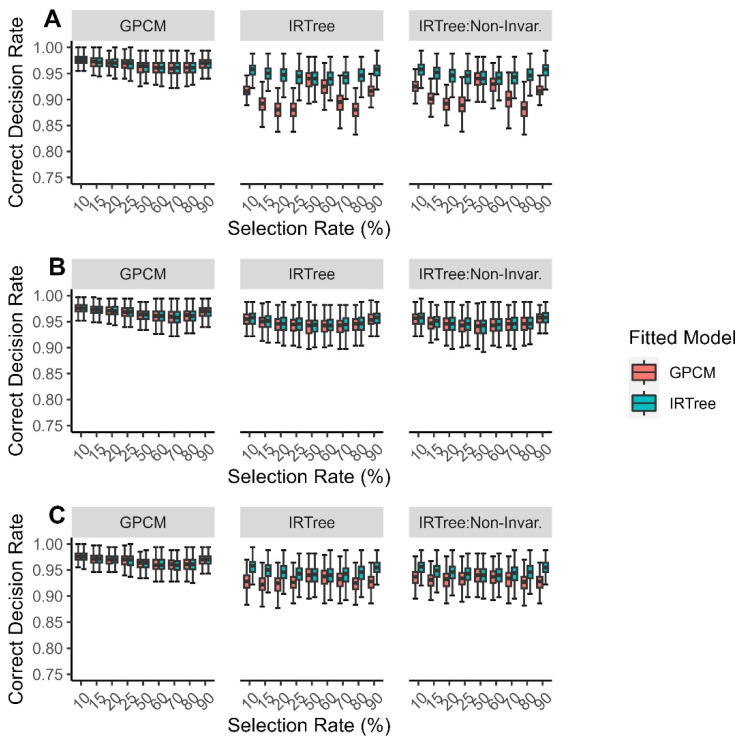
Comparison of fitted model correct decision rates for each data-generating model by (**A**) low levels of extreme response style; (**B**) moderate levels of extreme response style; (**C**) high levels of extreme response style.

The correct decision rates when the data were fitted with an IRTree model were much higher and more consistent across the generating model, selection rate, and level of extreme response style than when the data were fitted with a GPCM. When the data were generated with a GPCM, there was virtually no difference in the correct decision rates between the IRTree model and the GPCM and little variation across the selection rate. However, when the data were fitted with a GPCM and misspecified, the correct decision rates tended to be lower and to vary between the levels of extreme response style and selection rate. The differences tended to be clearer when the level of extreme response style was low, but for both the low and the high levels of extreme response style, the correct decision rate for the GPCM when misspecified was minimized when the selection rate was either 20% or 80% and maximized when the selection rate was 50%. Boxplots for the correct decision rates by selection rate and level of extreme response style are given in Figure 4.

**Figure 5:**
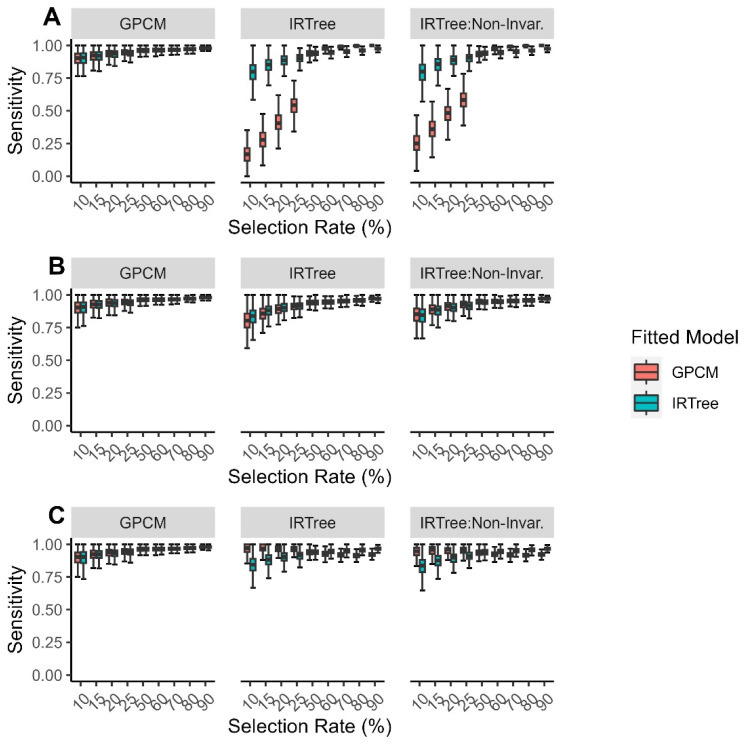
Comparison of fitted model sensitivity for each data-generating model by (**A**) low levels of extreme response style; (**B**) moderate levels of extreme response style; (**C**) high levels of extreme response style.

The sensitivity when the data were fitted with an IRTree model was more consistent across the generating model, selection rate, and level of extreme response style than when the data were fitted with a GPCM. When the data were fitted with a GPCM and misspecified, the sensitivity tended to be lower when the extreme response style and selection rate were low or when the extreme response style and selection rate were high. As shown in Figure 5, in situations where there is evidence of extreme response styles and the decision is highly selective, so only a small proportion of examinees are selected, we see the greatest differences between the sensitivity of the models for the low levels of extreme response style. This can be interpreted as the GPCM having a reduced ability to detect those with high levels of the content trait correctly when the extreme response style is low, while the IRTree model can detect those with high levels of content trait that should be selected regardless of the level of extreme response style.

**Figure 6:**
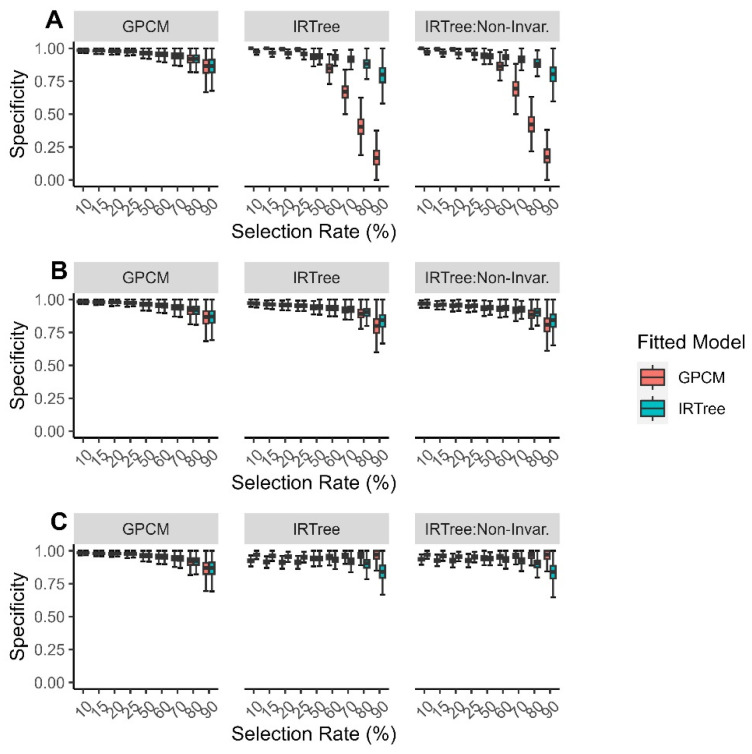
Comparison of fitted model specificity for each data-generating model by (**A**) low levels of extreme response style; (**B**) moderate levels of extreme response style; (**C**) high levels of extreme response style.

The specificity when the data were fitted with an IRTree model was much more consistent across the generating model, selection rate, and level of extreme response style than when fitted with the GPCM. However, when the data were fitted with a GPCM and misspecified, the specificity tended to vary between the levels of extreme response style and the selection rate. When the GPCM was misspecified, the specificity tended to be high when the extreme response style and selection rate were both low and when the extreme response style and selection rate were both high. As shown in Figure 6, in the situations where there is evidence of extreme response styles and the decision is less selective, so a large proportion of examinees are selected, we see the greatest differences between the specificity of the models for the low levels of extreme response style. This can be interpreted as the GPCM having an increased tendency to incorrectly identify those with low levels of the content trait when the extreme response style is also low, while the IRTree model demonstrates consistent accuracy when identifying low performers regardless of the level of extreme response style.

**Figure 7:**
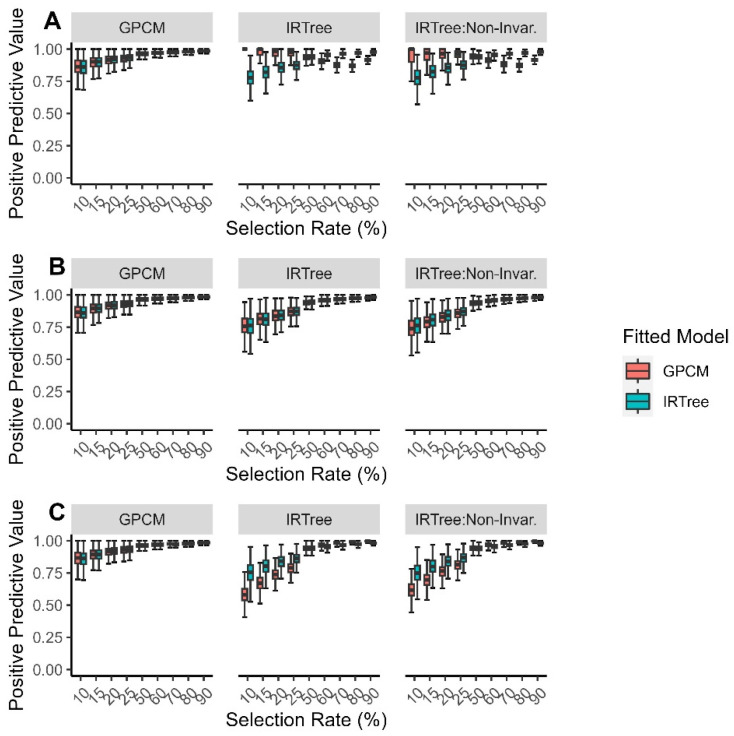
Comparison of fitted model positive predictive values for each data-generating model by (**A**) low levels of extreme response style; (**B**) moderate levels of extreme response style; (**C**) high levels of extreme response style.

The positive predictive values when the data were fitted with an IRTree model were more consistent across the generating model, selection rate, and level of extreme response style than when the data were fitted with a GPCM. When the data were fitted with a GPCM and misspecified, the positive predictive values tended to be high when the extreme response style and selection rate were both low. The positive predictive values tended to be low when the selection rate was high and the extreme response style was low or when the extreme response style was high and the selection rate was low (Figure 7). 

**Figure 8:**
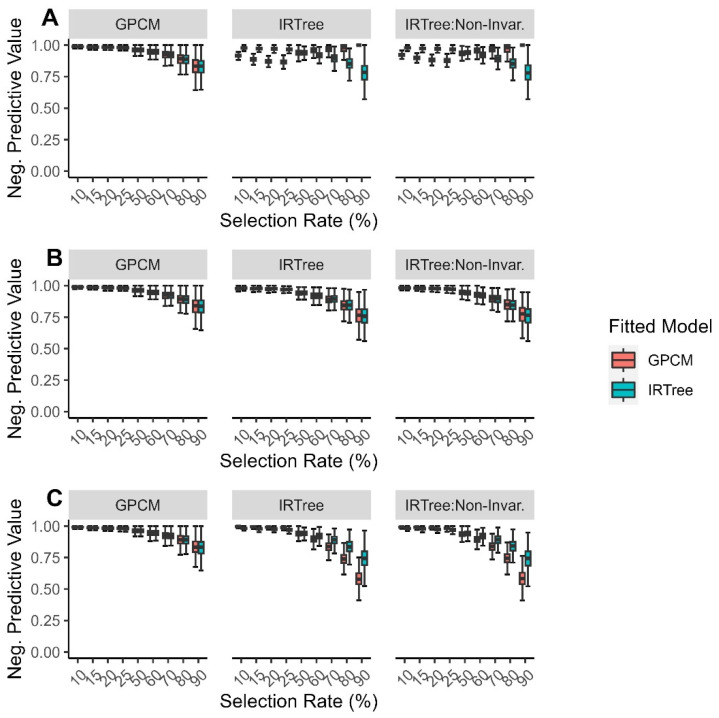
Comparison of fitted model negative predictive values for each data-generating model by (**A**) low levels of extreme response style; (**B**) moderate levels of extreme response style; (**C**) high levels of extreme response style.

The negative predictive values when the data were fitted with an IRTree model were more consistent across the generating model, selection rate, and level of extreme response style than when the data were fitted with a GPCM. When the data were fitted with a GPCM and misspecified, the negative predictive values tended to be lower when the levels of the extreme response style and selection rate were both low or when the extreme response style and selection rate were both high (Figure 8). 

**Figure 9:**
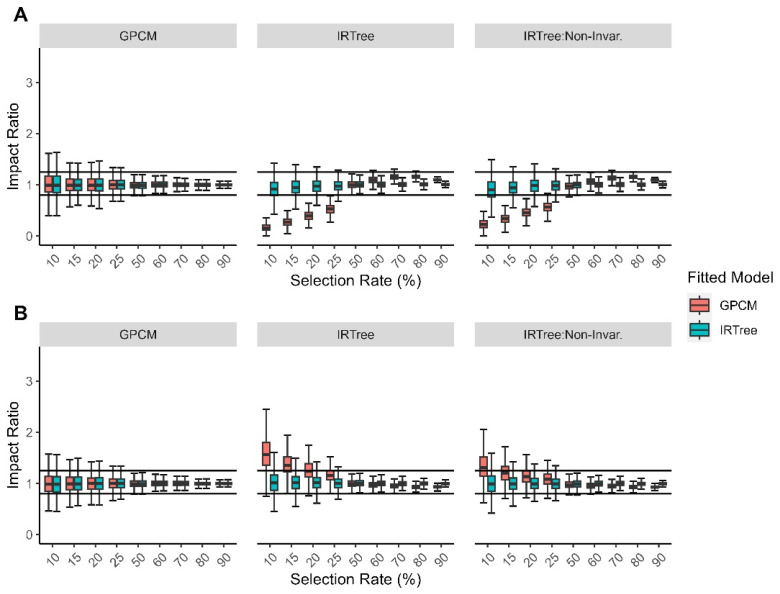
Comparison of fitted model impact ratios for each data-generating model by (**A**) low levels of extreme response style; (**B**) high levels of extreme response style.

We chose the moderate level of the extreme response style group to be the reference group for calculating adverse impact. In general, we found that those with high levels of extreme response style were selected at higher rates than those with a moderate level of extreme response style in the cases where the data were generated with an IRTree model, with or without directional invariance. Likewise, those with low levels of extreme response style were selected at lower rates than those with moderate levels of extreme response style. When the data were generated with a GPCM, the extreme response style trait was not used in generating the responses; so, the observed selection rates were equal across the levels of extreme response style in this case, and there were rarely instances of adverse impact. When the data were generated with an IRTree model, the extreme response style trait did impact the item responses. When the models were fitted to the IRTree generated data (with or without directional non-invariance), we found that the IRTree model generally demonstrated more acceptable impact ratios across all levels of the selection rate condition. However, when the IRTree generated data were fitted with the GPCM, we found far more instances of adverse impact for those with both high and low levels of extreme response style when the selection rate condition was below 50%. While there was still fluctuation in the impact ratios for the GPCM fitted data at a rate which was above the selection rate condition of 50%, the observations generally fell within the acceptable range of [0.80, 1.25]. Regardless of the generating model, the IRTree model displays very similar impact ratios across the selection rate and level of extreme response style, suggesting that the IRTree model is less susceptible to making decisions that result in adverse impact in cases of model misspecification. Boxplots for the impact ratio by selection rate and level of extreme response style are given in Figure 9.

## 4. Application

To investigate the applicability of our findings to real-world examples, data were collected from the Open-Source Psychometrics Project Fisher Temperament Inventory raw dataset (N=4967). The Fisher Temperament Inventory is a four-category Likert-type personality scale measuring four temperaments (Curious/Energetic, Cautious/Social Norm Compliant, Analytical/Tough-minded, and Prosocial/Empathetic) associated with a unique neural system (dopamine, serotonin, testosterone, and estrogen/oxytocin) (Fisher et al. 2010).

### 4.1. Design

After data cleaning, our sample size was N=3265. Of those in the sample, 29.98% identified as male, 68.06% identified as female, and 1.96% identified as other. The mean age of the sample was 33.57 (SD = 12.45), with ages ranging from 18 to 78. Of the participants, 10.08% identified as Asian, 0.70% identified as Arab, 2.24% identified as Black, 0.83% identified as Native American, 77.52% identified as White, and 8.64% identified as other. Furthermore, 1.29% reported completing less than a high school education, 23.88% reported completing high school, 43.50% reported completing a university degree, and 31.42% reported completing a graduate degree. 

We conducted a confirmatory factor analysis to ensure that our data followed a similar factor loading structure to the findings of the original Fisher Temperament Inventory (Fisher et al. 2010). We found that a four-factor structure was sufficient under an oblimin factor rotation, $\chi^{2}\left(1322\right)=15,511.31$, RMSEA=0.057, 95% CI [0.056,0.058], p<.05. Due to prior evidence of concurrent validity between the Curious/Energetic dimension of the FTI and the Extraversion factor of the five-factor personality inventory (Fisher et al. 2015), we chose to use this as our content trait of interest and selected these items as our responses for the selection procedure.

For the analysis, each person had three estimated trait scores: the content trait score estimated by the GPCM, the content trait score estimated by the IRTree model, and the extreme response style score estimated by the IRTree model. As both traits were estimated by the IRTree model in a manner like that of a bifactor model, for identifiability purposes the latent traits were assumed to be orthogonal. The extreme response style score was then categorized as low (bottom third), moderate (middle third), or high (top third). Using the same levels for the selection rate as the simulation, the top percentages of the content trait scores were then chosen to be “selected” to mimic being selected to move forward in a hypothetical applicant selection process.

AIC and BIC statistics were used to compare the model fit between the GPCM and the IRTree model. We also determined the agreement rates of the GPCM and the IRTree indicated selection decision for each selection rate. Then, a paired samples t-test to evaluate the differences between the scores estimated by the GPCM and the IRTree model was conducted. We then calculated Kendall’s rank-order correlation between the GPCM and IRTree estimated scores, as well as the Pearson’s correlation between each of the continuous variables. Next, the demographic group means for each estimated score were compared. Finally, the demographic group impact ratios by scoring model and selection rate condition were compared. To better depict the similarities and differences between the impact ratios of the two models and to include a measure of effect size, we examine here the log transformations of the impact ratio for each model, where log($IR_{T}$) is the log transformation of the impact ratio for the IRTree model and log($IR_{G}$) is the log transformation of the impact ratio for the GPCM. To calculate an effect size for these differences, we use a measure of standard error associated with risk ratios. Consider a contingency table demonstrating the “risk” of being selected depending on whether an examinee is a member of the focal group or the reference group (Table 2).

To estimate the standard error of the impact ratio for a model, we use the following formula:

(5) $$\hat{\sigma }=\;\sqrt{\left(\frac{1}{n_{11}}+\frac{1}{n_{21}}\right)-\left(\frac{1}{n_{11}+n_{12}}+\frac{1}{n_{21}+n_{22}}\right)},$$

After estimating the standard error, we calculate the effect size for the difference in log impact ratio between the IRTree model and the GPCM as:

(6) $$\hat{\delta }=\;\frac{log(lR_{T})-log(IR_{G})}{\hat{\sigma }},$$

Under this transformation, a log impact ratio of 0 means there is no difference in the selection rates between the focal and reference group, and values exceeding ±.25 are considered evidence of adverse impact. In line with similar effect size thresholds, we use benchmarks for small (0.20) moderate (0.50) and large (0.80) effects (Cohen 1988).

### 4.2. Results

#### 4.2.1. Correlations

All correlations are reported in Table 3. For the fitted models, the GPCM considered all the item responses to be indicative of a single underlying content trait, $\theta_{C,GPCM}$; the IRTree model considered the pseudo-item responses for the first node to be indicative of a single underlying content trait, $\theta_{C,IRTree}$, and the pseudo-item responses for the second and third nodes to be indicative of a common underlying extreme response style trait, $\theta_{ERS,IRTree}$. The extreme response style score had a stronger correlation with the GPCM estimated score than with the IRTree estimated score. This makes sense, as the IRTree separates out the extreme response style trait from the content trait, while the GPCM does not. This strength of relationship between the extreme response style and the estimated content score has been found in prior research to potentially increase bias (Plieninger 2017). Age was found to have a small negative correlation with extreme response style, a smaller negative correlation with the GPCM estimated score, and an even smaller negative correlation with the IRTree estimated score. This finding is supported by previous research, as several studies have found a relationship between age and extreme response style (Batchelor and Miao 2016).

#### 4.2.2. Model Fit and Scoring Comparison

The model fit statistics are provided in Table 4. The IRTree model was found to have better overall fit than the GPCM, as shown by smaller values of AIC and BIC for the IRTree model. As the IRTree model demonstrates a better fit, we can conclude that there is evidence of extreme response styles in these data. To determine the level of agreement between the rankings of the GPCM and the IRTree scores, we calculated the Kendall rank correlation. The GPCM estimated rankings and the IRTree estimated rankings had a strong, positive correlation (τ=.91, z=77.80, p<.001). This suggests that there were not many discordant pairs; the scores were ranked fairly similarly between the two models. As the rankings between the models were highly similar, there was a relatively high rate of agreement in the selection decisions between the two models. The highest agreement rate occurred when the selection rate was set to 70% (98.90% agreement), and the lowest agreement rate occurred when the selection rate was set to 25% (93.51% agreement). A paired samples t-test indicated that while the scores from the GPCM (M=0.00, SD=0.91) and the IRTree model (M=−0.02, SD=0.87) were significantly different from one another ($t\left(3264\right)=-5.83$, p<.001), the Cohen’s deffect size was negligible, d=−0.02, 95% CI [−0.07, 0.03]. The improvement in model fit in terms of accounting for response styles and the small change in parameter estimates are both consistent with prior research (Plieninger 2017). The differences between the model content trait estimates seem to be the most exaggerated at the extreme ends of the score, and they also have a relationship with the level of the extreme response style trait, as estimated by the IRTree model.

#### 4.2.3. Analysis of Variance

For each score (GPCM estimated content trait, IRTree estimated content trait, IRTree estimated extreme response style), analysis of variance tests were conducted to determine whether there were demographic differences in the estimated scores, as estimated by each model. A separate test was run for each categorical demographic variable and each score. In instances where an omnibus test indicated the presence of group differences, we conducted pairwise post hoc analyses using a Tukey adjustment to identify specific group differences. To accommodate unequal sample sizes across groups, Satterthwaite degrees of freedom were used. The findings of the post hoc analyses are reported below. The first analysis compared the GPCM estimated content trait scores across the groups. The second analysis compared the IRTree estimated content trait scores across the groups. The third analysis compared the IRTree estimated extreme response style scores across the groups.

We did not find significant pairwise differences in the GPCM estimated content score by gender. There was evidence for significant differences in the GPCM estimated content score by race. The Asian participants had a significantly higher level of content trait than the White participants ($t\left(3254\right)=4.03$, p<.001), as did the Arab participants ($t\left(3254\right)=3.28$, p<.05) and Black participants ($t\left(3254\right)=2.856$, p<.05). There was also evidence for significant differences in the GPCM estimated content score by level of education. The participants with a high school diploma demonstrated a significantly lower level of the GPCM estimated content trait than those with a university degree ($t\left(1532.1\right)=-3.14$, p<.01) and those with a graduate degree ($t\left(1562.4\right)=-2.98$, p<.05).

The findings were very similar for the IRTree estimated content score. We did not find significant pairwise differences in the IRTree estimated content score by gender. There was evidence for significant differences in the IRTree estimated content score by race. The Asian participants had a significantly higher level of content trait than the White participants ($t\left(468.0\right)=3.77$, p<.01), as did the Arab participants ($t\left(21.9\right)=3.14$, p<.05), when estimated with the IRTree model. However, there was not a significant difference in the content scores for the Black and White participants when the IRTree was fitted to the data compared to when the GPCM was used. Additionally, we found evidence for significant differences in the IRTree estimated content score by level of education. The participants that had completed high school had a significantly lower level of content trait than the participants with a university degree ($t\left(1322.3\right)=-3.26$, p<.01) or a graduate degree ($t\left(1497.4\right)=-3.56$, p<.01), when estimated with an IRTree model.

The extreme response style was only estimated using the IRTree model. There was evidence for significant differences in the extreme response style by gender and level of education. The male participants had a significantly higher level of extreme response style than the female participants ($t\left(1779.0\right)=5.44$, p<.001). The participants with a high school diploma had a significantly higher level of extreme response style than the participants with a university degree ($t\left(1512.8\right)=3.89$, p<.001) and the participants with a graduate degree ($t\left(1567.2\right)=5.443$, p<.001). Interestingly, there were not significant pairwise differences in the extreme response style by race in this dataset. While prior research has typically found significant differences in the extreme response style by race, our findings for the extreme response style differences across gender and levels of education are consistent with the prior research (Batchelor and Miao 2016).

#### 4.2.4. Adverse Impact

**Figure 10:**
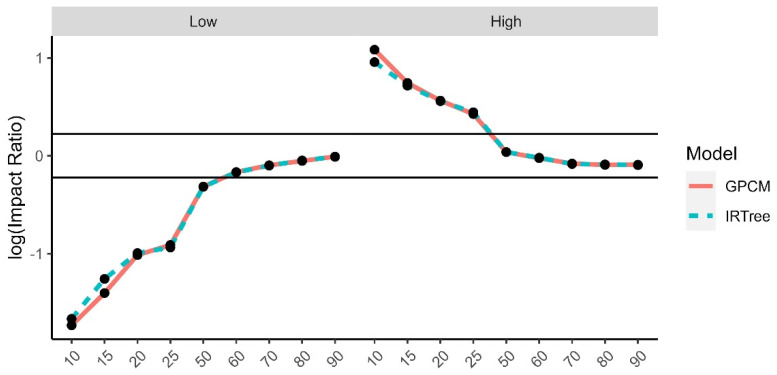
Comparison of fitted model impact ratios across levels of extreme response style, using moderate levels of extreme response style as the reference group. Effect sizes ranged from −1.03 to 0.80.

**Figure 11:**
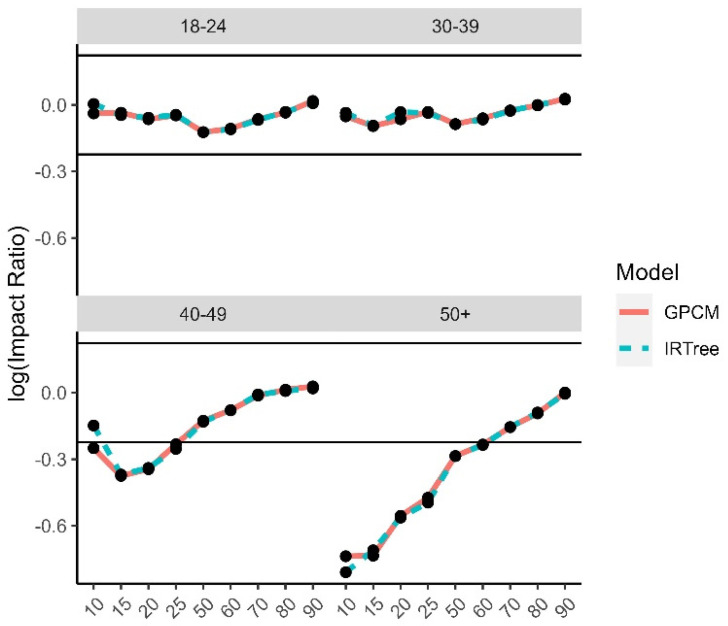
Comparison of fitted model impact ratios across age groups, using 25–29 as the reference group. Effect sizes ranged from −0.55 to 0.55.

**Figure 12:**
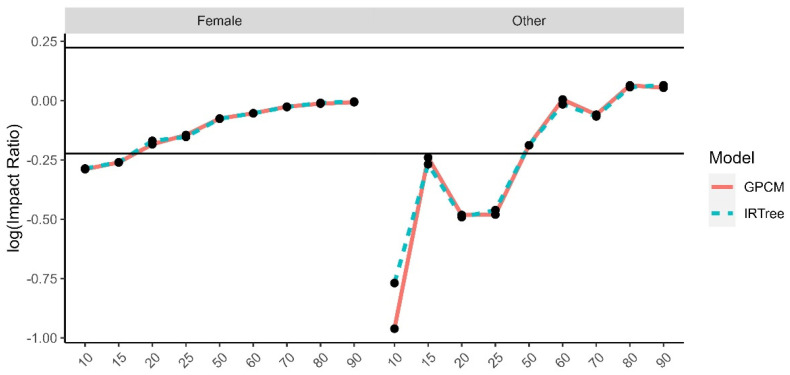
Comparison of fitted model impact ratios across gender, using male as the reference group. Effect sizes ranged from −0.20 to 0.36.

**Figure 13:**
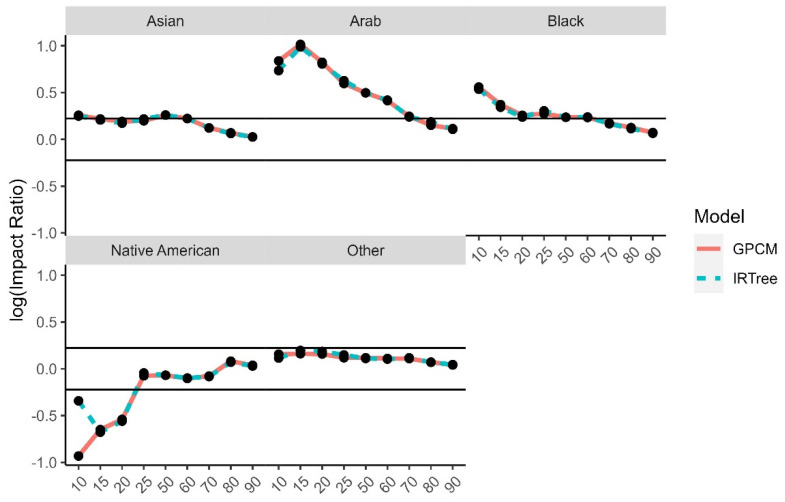
Comparison of fitted model impact ratios across race, using White as the reference group. Effect sizes ranged from −1.08 to 0.60.

**Figure 14:**
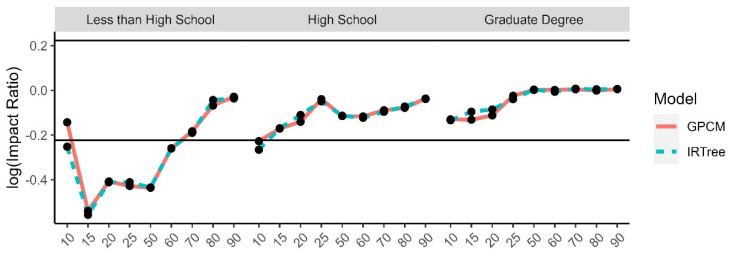
Comparison of fitted model impact ratios across levels of education, using university degree as the reference group. Effect sizes ranged from −0.28 to 0.37.

The impact ratios were calculated as the selection rate of a minority group divided by the selection rate of the reference group for each demographic trait and level of extreme response style and then log transformed. The reference groups were selected to be White for race, male for gender, 24–29 for age, university degree for level of education, and moderate level of extreme response style for the comparison by level of extreme response style. There is a high degree of overlap between the impact ratios for each model, due to the high rate of agreement and rank-order similarities between the two model-estimated content trait scores. While the differences were small between the models, even some small differences were found to have small to moderate effect sizes. The graphs for the log impact ratios and the corresponding effect size differences for the level of extreme response style (Figure 10), age group (Figure 11), gender (Figure 12), race (Figure 13), and level of education (Figure 14) are provided.

## 5. Discussion

IRTree models are a flexible family of item response models that can be modified to align with a theorized response process. The purpose of our study was to compare the selection validity and adverse impact reduction capabilities of an IRTree model that follows a theorized response process with a GPCM that does not follow such a process. First, we ran a Monte Carlo simulation with varying sample sizes and test lengths. The responses were generated according to one of three data-generating models (GPCM, IRTree, and IRTree with directional non-invariance), then fitted according to both models of interest (GPCM and IRTree). The selection decisions were made at a series of selection rate cutoffs. We collected model fit statistics, correct decision rate, sensitivity, specificity, positive predictive value, negative predictive value, and impact rate by level of extreme response style (low, moderate, and high) for each combination of data-generating model and fitted model.

When the response style effects were present (i.e., the data were generated via an IRTree model), not accounting for these effects (i.e., misspecifying the model as a GPCM) led to poor selection validity and low impact ratios. However, when the response style effects were not present—that is, the data were generated via a GPCM—misspecifying the model as IRTree to account for these non-existent effects had very little detrimental effect on selection validity. As such, we conclude that in instances where extreme response styles may or may not be present, an IRTree model can be used to help maintain high selection validity. We also found that using an IRTree model has the potential to reduce the adverse impact that may occur as a result of extreme response styles. Both models seem to be fairly robust with regard to problems of directional non-invariance on the item level.

Following the investigation into the selection validity of the two models, we applied both models to an example dataset. We used responses to the Fisher Temperament Inventory, available on the Open-Source Psychometric Project raw data website, to test the practical applicability of our findings from the simulation. We fitted both a GPCM and an IRTree model, estimated the content trait levels, and made selection decisions based on content trait score. Using the IRTree model, we also estimated the levels of extreme response style for each person. The IRTree model demonstrated a better fit, suggesting that there was evidence of extreme response styles in these data. There were slight changes in the content trait estimates between the models, but the agreement between the two models was high and consistent with prior research (Plieninger 2017). We found that the content trait scores were related to demographic traits (e.g., race, education, and age) regardless of the fitted model. There were no significant pairwise differences in content traits by gender for either model. The impact ratios were also consistent between the fitted models.

### 5.1. Contributions

In this article, we provide several unique contributions to the study of IRTree models. First, we found the IRTree model to be less susceptible to reduced selection validity in cases of model misspecification than the GPCM. When the extreme response styles are evident in the data, the IRTree models allow for higher selection validity. Even when extreme response styles are not evident in the data, the IRTree models offer only slightly less validity than a correctly specified GPCM. However, the reverse is not true: when the extreme response styles are present in the data and a model is used that does not account for these effects (e.g., GPCM), the selection validity suffers and the incidence of adverse impact increases.

Second, we demonstrated the potential effect of not accounting for extreme response styles in the data on adverse impact. In the simulation, we found that when there was evidence of extreme response styles, the impact ratios typically fell within the acceptable range when the IRTree model was fitted to the data; however, the impact ratios varied drastically for groups with low or high levels of extreme response style when a GPCM was fitted.

Third, we found that an IRTree model did demonstrate a better fit than a GPCM in a real dataset. There was evidence of extreme response styles in this dataset.

Finally, we found that while the IRTree model does account for potential noise and can improve selection validity, the impact of model selection and model misspecification likely depends on the relationship of the noise trait and the content trait of interest. In situations where the noise trait (i.e., extreme response style) has a relationship with the content trait (i.e., personality trait), the adverse impact may only differ to the degree that a better fitting model would improve the accuracy of the model estimates by reducing the noise in the content trait estimation, as was the case in the applied example. The finding that response styles may have a relatively small impact on parameter estimates is supported by prior research (Schimmack et al. 2002; Plieninger 2017); however, our study extends these findings to encompass selection decisions and adverse impact.

### 5.2. Limitations and Future Directions

Our study had several limitations. Given the small effect that the extreme response style has on the overall response pattern, there may not have been large enough subsets to have the necessary statistical power to estimate so many parameters. We suggest future research to determine the sample size required for each additional node of parameters estimated beyond the initial node.

In our study, only the IRTree model was parameterized to estimate a separate latent trait for the extreme response style. However, some have proposed adaptations to the GPCM to account for extreme response style (Jin and Wang 2014). Future studies may wish to compare an adapted GPCM to an IRTree model to differentiate between the effect of accounting for extreme response style and the effect of model structure.

In the simulation, we were primarily interested in comparing the validity of an IRTree model to a GPCM when directional invariance at the item level may or may not exist. We assume that the researcher does not have knowledge; so, the IRTree model we use does not estimate any additional parameters that that might account for directional non-invariance. The IRTree model may demonstrate even greater fit if parameters were to be estimated that specifically did account for directional non-invariance, but this line of investigation fell outside the scope of the current research.

Finally, while our study examined the GPCM and an IRTree model with 2PL models at each node, the IRTree model has the potential for much more flexibility than we demonstrate here. Future research may also extend the findings here to IRTree models with 3PL, 4PL, or polytomous IRT models at each node, or it may consider more highly parameterized models (i.e., nominal response model) for a basis of comparison.

In a much broader sense, there is work to be carried out regarding IRTree models in real world contexts and the numerous potential applications of the knowledge gained from these models. Applied research could consider simplified scoring methods using the IRTree response process structure to make this approach more accessible for organizations. The information gained from this family of models could be used to detect the impact of extreme response styles during a trial run for an experiment, allowing the opportunity for researchers to adjust the item format if necessary.

## 6. Conclusions

In conclusion, the IRTree works well in accounting for potential sources of noise in Likert-type data. In situations where extreme response style may be at play, the models that do not account for this additional noise may be at an increased risk of instances of adverse impact. Through simulation, we demonstrate that the IRTree model has the potential to increase selection validity and reduce instances of adverse impact in situations where response styles may be at play, without much loss in selection validity when the model is misspecified. In practical settings, where little may be known about the underlying response process for a Likert scale, particularly when extreme response styles are suspected, we recommend considering the IRTree model to preserve selection validity and reduce the risk of adverse impact due to response style influence. Though there may only be subtle influences of response styles in many practical settings, the IRTree model can be thought of as a flexible and general-purpose model that has straightforward interpretability, high levels of selection validity under a variety of conditions, and the potential to improve model fit.

## Figures and Tables

**Table 1:** Node responses for extreme response style IRTree pseudo-item mapping convention.

	Node Response		
Selected Response	$Y_{ij1}^{*}$	$Y_{ij2}^{*}$	$Y_{ij3}^{*}$
$Y_{ij}=1$	0	1	NA
$Y_{ij}=2$	0	0	NA
$Y_{ij}=3$	1	NA	0
$Y_{ij}=4$	1	NA	1

**Table 2:** Risk ratio contingency table.

	Selected	Not Selected
Focal Group	$n_{11}$	$n_{12}$
Reference Group	$n_{21}$	$n_{22}$

**Table 3:** Pearson’s *r* correlations between GPCM content score, IRTree estimated scores, and age.

	$\theta_{C,GPCM}$	$\theta_{C,IRTree}$	$\theta_{ERS,IRTree}$	Age
$\theta_{C,GPCM}$	--			
$\theta_{C,IRTree}$	.98	--		
$\theta_{ERS,IRTree}$	.15	.05	--	
Age	−.06	−.05	−.16	*--*

Note: All correlations are significant, *p* < .05.

**Table 4:** Model fit statistics.

	AIC	BIC
IRTree	97,029.28	97,711.48
GPCM	98,867.81	99,208.90

## Data Availability

The data used for the applied example can be found at http://openpsychometrics.org/_rawdata/FTI-data.zip, accessed on 19 June 2022.

## Supplementary Materials

The following supporting information can be downloaded at: https://www.mdpi.com/article/10.3390/jintelligence11110216/s1, Table S1: Simulation Results: *N* = 500, *n* = 25, Table S2: Simulation Results: *N* = 500, *n* = 50, Table S3: Simulation Results: *N* = 1000, *n* = 25, Table S4: Simulation Results: *N* = 1000, *n* = 50, Table S5: Simulation Results: *N* = 2000, *n* = 25, Table S6: Simulation Results: *N* = 2000, *n* = 50.
