# Supplementary material for: Using IRTree Models to Promote Selection Validity in the Presence of Extreme Response Styles
Source: J Intell. 2023 Nov 17;11(11):216. doi: 10.3390/jintelligence11110216 (PMC10672242; doi:10.3390/jintelligence11110216)
Supplement: Supplementary file 1 [file jintelligence-11-00216-s001.zip › jintelligence-2468861-supplementary.pdf]

Supplementary Materials

Table S1. Simulation Results, N = 500, n = 25.

Sel. Rate	Gen. Mod.	Fit Mod.	Correct Decision Rate		Sensitivity		Specificity		Positive Predictive Value		Negative Predictive Value	
			M	SD	M	SD	M	SD	M	SD	M	SD
0.1	GPCM	GPCM	0.97	0.01	0.88	0.05	0.98	0.01	0.83	0.04	0.99	0.01
		IRTree	0.97	0.01	0.87	0.04	0.98	0.01	0.83	0.04	0.99	0.00
	IRTree	GPCM	0.93	0.01	0.63	0.06	0.96	0.01	0.65	0.06	0.96	0.01
		IRTree	0.95	0.01	0.79	0.06	0.97	0.01	0.72	0.05	0.98	0.01
	IRTree:Non-Invar.	GPCM	0.93	0.01	0.67	0.07	0.96	0.01	0.67	0.06	0.96	0.01
		IRTree	0.95	0.01	0.79	0.05	0.97	0.01	0.72	0.04	0.98	0.01
0.15	GPCM	GPCM	0.97	0.01	0.91	0.03	0.98	0.01	0.87	0.04	0.98	0.01
		IRTree	0.97	0.01	0.90	0.04	0.98	0.01	0.88	0.04	0.98	0.01
	IRTree	GPCM	0.92	0.01	0.69	0.05	0.96	0.01	0.74	0.04	0.95	0.01
		IRTree	0.94	0.01	0.85	0.04	0.96	0.01	0.78	0.04	0.97	0.01
	IRTree:Non-Invar.	GPCM	0.92	0.01	0.72	0.05	0.96	0.01	0.74	0.05	0.95	0.01
		IRTree	0.94	0.01	0.85	0.04	0.96	0.01	0.78	0.04	0.97	0.01
0.2	GPCM	GPCM	0.96	0.01	0.93	0.03	0.97	0.01	0.90	0.03	0.98	0.01
		IRTree	0.96	0.01	0.92	0.03	0.97	0.01	0.90	0.03	0.98	0.01
	IRTree	GPCM	0.91	0.01	0.75	0.05	0.95	0.01	0.80	0.04	0.94	0.01
		IRTree	0.94	0.01	0.89	0.03	0.95	0.01	0.83	0.03	0.97	0.01
	IRTree:Non-Invar.	GPCM	0.91	0.01	0.77	0.05	0.95	0.01	0.80	0.04	0.94	0.01
		IRTree	0.94	0.01	0.88	0.04	0.95	0.01	0.82	0.03	0.97	0.01
0.25	GPCM	GPCM	0.96	0.01	0.93	0.02	0.97	0.01	0.92	0.03	0.98	0.01
		IRTree	0.96	0.01	0.93	0.02	0.97	0.01	0.92	0.03	0.98	0.01
	IRTree	GPCM	0.91	0.01	0.79	0.03	0.95	0.01	0.83	0.03	0.93	0.01
		IRTree	0.93	0.01	0.89	0.02	0.94	0.01	0.84	0.02	0.96	0.01
	IRTree:Non-Invar.	GPCM	0.91	0.01	0.81	0.03	0.95	0.01	0.84	0.03	0.94	0.01
		IRTree	0.93	0.01	0.89	0.03	0.95	0.01	0.85	0.03	0.96	0.01
0.5	GPCM	GPCM	0.96	0.01	0.95	0.02	0.96	0.01	0.96	0.01	0.95	0.02
		IRTree	0.95	0.01	0.95	0.02	0.96	0.02	0.96	0.01	0.95	0.02
	IRTree	GPCM	0.93	0.01	0.93	0.02	0.93	0.02	0.93	0.02	0.93	0.02
		IRTree	0.93	0.01	0.93	0.02	0.93	0.02	0.93	0.02	0.93	0.02
	IRTree:Non-Invar.	GPCM	0.93	0.01	0.93	0.02	0.93	0.02	0.93	0.02	0.93	0.02
		IRTree	0.93	0.01	0.93	0.02	0.93	0.02	0.93	0.02	0.93	0.02
0.6	GPCM	GPCM	0.95	0.01	0.96	0.01	0.95	0.02	0.97	0.01	0.94	0.02
		IRTree	0.95	0.01	0.96	0.01	0.95	0.02	0.97	0.01	0.94	0.02
	IRTree	GPCM	0.92	0.01	0.94	0.01	0.90	0.03	0.93	0.02	0.91	0.02
		IRTree	0.93	0.01	0.94	0.01	0.92	0.02	0.95	0.01	0.91	0.02
		GPCM	0.93	0.01	0.94	0.02	0.90	0.02	0.94	0.01	0.91	0.02

	IRTree:Non-Invar.	IRTree	0.93	0.01	0.94	0.02	0.92	0.02	0.95	0.01	0.91	0.02
0.7	GPCM	GPCM	0.95	0.01	0.96	0.01	0.93	0.02	0.97	0.01	0.91	0.02
		IRTree	0.95	0.01	0.96	0.01	0.93	0.03	0.97	0.01	0.91	0.02
	IRTree	GPCM	0.92	0.01	0.95	0.01	0.84	0.03	0.93	0.01	0.87	0.03
		IRTree	0.93	0.01	0.94	0.01	0.90	0.02	0.96	0.01	0.87	0.03
	IRTree:Non-Invar.	GPCM	0.91	0.02	0.95	0.01	0.84	0.04	0.93	0.01	0.87	0.03
		IRTree	0.93	0.01	0.94	0.01	0.91	0.03	0.96	0.01	0.87	0.02
0.8	GPCM	GPCM	0.95	0.01	0.96	0.01	0.90	0.03	0.98	0.01	0.86	0.03
		IRTree	0.95	0.01	0.96	0.01	0.90	0.03	0.98	0.01	0.86	0.03
	IRTree	GPCM	0.91	0.01	0.95	0.01	0.75	0.04	0.94	0.01	0.79	0.04
		IRTree	0.94	0.01	0.95	0.01	0.88	0.04	0.97	0.01	0.82	0.03
	IRTree:Non-Invar.	GPCM	0.91	0.01	0.95	0.01	0.75	0.04	0.94	0.01	0.80	0.04
		IRTree	0.94	0.01	0.95	0.01	0.88	0.03	0.97	0.01	0.82	0.03
0.9	GPCM	GPCM	0.96	0.01	0.98	0.01	0.84	0.05	0.98	0.01	0.80	0.05
		IRTree	0.96	0.01	0.98	0.01	0.84	0.05	0.98	0.01	0.80	0.05
	IRTree	GPCM	0.93	0.01	0.96	0.01	0.63	0.06	0.96	0.01	0.65	0.06
		IRTree	0.95	0.01	0.97	0.01	0.79	0.06	0.98	0.01	0.72	0.05
	IRTree:Non-Invar.	GPCM	0.93	0.01	0.96	0.01	0.64	0.06	0.96	0.01	0.66	0.07
		IRTree	0.95	0.01	0.97	0.01	0.80	0.05	0.98	0.01	0.73	0.05

Table S2. Simulation Results, N = 500, n = 50.

**Sel. Rate**	**Gen. Mod.**	**Fit Mod.**	**Correct Decision Rate**		**Sensitivity**		**Specificity**		**Positive Predictive Value**		**Negative Predictive Value**	
			**M**	**SD**	**M**	**SD**	**M**	**SD**	**M**	**SD**	**M**	**SD**
0.1	GPCM	GPCM	0.98	0.01	0.91	0.05	0.99	0.01	0.89	0.05	0.99	0.01
		IRTree	0.98	0.01	0.91	0.04	0.99	0.01	0.88	0.05	0.99	0.00
	IRTree	GPCM	0.94	0.01	0.65	0.07	0.97	0.01	0.69	0.07	0.96	0.01
		IRTree	0.96	0.01	0.85	0.05	0.98	0.01	0.80	0.05	0.98	0.01
	IRTree:Non-Invar.	GPCM	0.94	0.01	0.69	0.06	0.97	0.01	0.72	0.06	0.97	0.01
		IRTree	0.96	0.01	0.85	0.05	0.98	0.01	0.79	0.05	0.98	0.01
0.15	GPCM	GPCM	0.98	0.01	0.93	0.03	0.98	0.01	0.92	0.03	0.99	0.01
		IRTree	0.98	0.01	0.93	0.03	0.98	0.01	0.92	0.04	0.99	0.01
	IRTree	GPCM	0.92	0.01	0.70	0.05	0.96	0.01	0.77	0.06	0.95	0.01
		IRTree	0.96	0.01	0.88	0.04	0.97	0.01	0.84	0.04	0.98	0.01
	IRTree:Non-Invar.	GPCM	0.93	0.01	0.74	0.04	0.96	0.01	0.78	0.05	0.95	0.01
		IRTree	0.96	0.01	0.88	0.04	0.97	0.01	0.84	0.04	0.98	0.01
0.2	GPCM	GPCM	0.97	0.01	0.94	0.03	0.98	0.01	0.92	0.03	0.99	0.01
		IRTree	0.97	0.01	0.94	0.03	0.98	0.01	0.93	0.03	0.98	0.01
	IRTree	GPCM	0.92	0.01	0.76	0.04	0.96	0.01	0.82	0.04	0.94	0.01

		IRTree	0.95	0.01	0.91	0.03	0.97	0.01	0.87	0.03	0.98	0.01
	IRTree:Non-Invar.	GPCM	0.93	0.01	0.78	0.04	0.96	0.01	0.83	0.04	0.95	0.01
		IRTree	0.95	0.01	0.91	0.03	0.96	0.01	0.87	0.03	0.98	0.01
0.25	GPCM	GPCM	0.97	0.01	0.95	0.02	0.98	0.01	0.94	0.02	0.98	0.01
		IRTree	0.97	0.01	0.94	0.02	0.98	0.01	0.95	0.02	0.98	0.01
	IRTree	GPCM	0.92	0.01	0.81	0.04	0.96	0.01	0.87	0.03	0.94	0.01
		IRTree	0.95	0.01	0.92	0.02	0.96	0.01	0.89	0.03	0.97	0.01
	IRTree:Non-Invar.	GPCM	0.93	0.01	0.83	0.04	0.96	0.01	0.88	0.03	0.94	0.01
		IRTree	0.95	0.01	0.92	0.03	0.96	0.01	0.89	0.03	0.97	0.01
0.5	GPCM	GPCM	0.97	0.01	0.97	0.02	0.97	0.02	0.97	0.02	0.97	0.01
		IRTree	0.97	0.01	0.97	0.01	0.97	0.01	0.97	0.01	0.97	0.01
	IRTree	GPCM	0.95	0.01	0.95	0.01	0.95	0.02	0.95	0.02	0.95	0.01
		IRTree	0.95	0.01	0.95	0.02	0.95	0.02	0.95	0.02	0.95	0.01
	IRTree:Non-Invar.	GPCM	0.95	0.01	0.95	0.02	0.95	0.02	0.95	0.02	0.95	0.02
		IRTree	0.95	0.01	0.95	0.02	0.95	0.02	0.95	0.01	0.95	0.01
0.6	GPCM	GPCM	0.97	0.01	0.97	0.01	0.96	0.02	0.97	0.01	0.95	0.02
		IRTree	0.97	0.01	0.97	0.01	0.96	0.02	0.97	0.01	0.95	0.02
	IRTree	GPCM	0.94	0.01	0.96	0.01	0.92	0.02	0.95	0.01	0.94	0.02
		IRTree	0.95	0.01	0.96	0.02	0.95	0.02	0.96	0.01	0.93	0.02
	IRTree:Non-Invar.	GPCM	0.94	0.01	0.96	0.02	0.92	0.02	0.95	0.02	0.94	0.02
		IRTree	0.95	0.01	0.96	0.01	0.94	0.02	0.96	0.01	0.93	0.02
0.7	GPCM	GPCM	0.96	0.01	0.97	0.01	0.95	0.02	0.98	0.01	0.93	0.03
		IRTree	0.96	0.01	0.97	0.01	0.95	0.02	0.98	0.01	0.93	0.02
	IRTree	GPCM	0.93	0.01	0.96	0.01	0.86	0.03	0.94	0.01	0.90	0.02
		IRTree	0.95	0.01	0.96	0.01	0.93	0.02	0.97	0.01	0.91	0.02
	IRTree:Non-Invar.	GPCM	0.93	0.01	0.96	0.01	0.86	0.03	0.94	0.01	0.91	0.02
		IRTree	0.95	0.01	0.96	0.01	0.93	0.02	0.97	0.01	0.91	0.02
0.8	GPCM	GPCM	0.97	0.01	0.98	0.01	0.93	0.03	0.98	0.01	0.91	0.03
		IRTree	0.97	0.01	0.98	0.01	0.93	0.03	0.98	0.01	0.91	0.03
	IRTree	GPCM	0.92	0.01	0.96	0.01	0.76	0.04	0.94	0.01	0.83	0.04
		IRTree	0.95	0.01	0.97	0.01	0.91	0.03	0.98	0.01	0.87	0.03
	IRTree:Non-Invar.	GPCM	0.92	0.01	0.96	0.01	0.76	0.04	0.94	0.01	0.83	0.04
		IRTree	0.96	0.01	0.97	0.01	0.91	0.03	0.98	0.01	0.87	0.03
0.9	GPCM	GPCM	0.97	0.01	0.98	0.01	0.89	0.05	0.99	0.01	0.86	0.05
		IRTree	0.97	0.01	0.98	0.01	0.89	0.05	0.99	0.01	0.86	0.05
	IRTree	GPCM	0.93	0.01	0.97	0.01	0.65	0.06	0.96	0.01	0.68	0.06
		IRTree	0.96	0.01	0.98	0.01	0.85	0.06	0.98	0.01	0.79	0.05
	IRTree:Non-Invar.	GPCM	0.94	0.01	0.97	0.01	0.66	0.06	0.96	0.01	0.70	0.05
		IRTree	0.96	0.01	0.98	0.01	0.84	0.06	0.98	0.01	0.79	0.04

Table S3. Simulation Results, N = 1000, n = 25.

Sel. Rate	Gen. Mod.	Fit Mod.	Correct Decision Rate		Sensitivity		Specificity		Positive Predictive Value		Negative Predictive Value	
			M	SD	M	SD	M	SD	M	SD	M	SD
0.1	GPCM	GPCM	0.97	0.01	0.89	0.04	0.98	0.00	0.84	0.03	0.99	0.00
		IRTree	0.97	0.01	0.89	0.04	0.98	0.00	0.84	0.04	0.99	0.00
	IRTree	GPCM	0.93	0.01	0.64	0.04	0.96	0.01	0.65	0.04	0.96	0.00
		IRTree	0.95	0.01	0.80	0.04	0.97	0.01	0.73	0.04	0.98	0.00
	IRTree:Non-Invar.	GPCM	0.93	0.01	0.67	0.04	0.96	0.01	0.67	0.04	0.96	0.00
		IRTree	0.95	0.01	0.80	0.05	0.97	0.01	0.73	0.03	0.98	0.01
0.15	GPCM	GPCM	0.97	0.01	0.91	0.02	0.98	0.01	0.87	0.02	0.98	0.00
		IRTree	0.97	0.01	0.91	0.02	0.98	0.01	0.88	0.03	0.98	0.00
	IRTree	GPCM	0.92	0.01	0.70	0.04	0.96	0.01	0.74	0.04	0.95	0.01
		IRTree	0.94	0.01	0.85	0.03	0.96	0.01	0.78	0.03	0.97	0.01
	IRTree:Non-Invar.	GPCM	0.92	0.01	0.73	0.03	0.96	0.01	0.75	0.04	0.95	0.01
		IRTree	0.94	0.01	0.85	0.03	0.96	0.01	0.78	0.03	0.97	0.01
0.2	GPCM	GPCM	0.96	0.01	0.93	0.02	0.97	0.01	0.90	0.02	0.98	0.00
		IRTree	0.96	0.01	0.93	0.02	0.97	0.01	0.90	0.02	0.98	0.00
	IRTree	GPCM	0.91	0.01	0.75	0.03	0.95	0.01	0.79	0.03	0.94	0.01
		IRTree	0.94	0.01	0.88	0.02	0.95	0.01	0.82	0.02	0.97	0.01
	IRTree:Non-Invar.	GPCM	0.91	0.01	0.77	0.03	0.95	0.01	0.80	0.03	0.94	0.01
		IRTree	0.94	0.01	0.88	0.02	0.95	0.01	0.82	0.02	0.97	0.01
0.25	GPCM	GPCM	0.96	0.01	0.94	0.02	0.97	0.01	0.91	0.02	0.98	0.01
		IRTree	0.96	0.01	0.94	0.02	0.97	0.01	0.92	0.02	0.98	0.01
	IRTree	GPCM	0.91	0.01	0.80	0.03	0.95	0.01	0.84	0.02	0.93	0.01
		IRTree	0.93	0.01	0.90	0.02	0.95	0.01	0.85	0.02	0.96	0.01
	IRTree:Non-Invar.	GPCM	0.92	0.01	0.81	0.03	0.95	0.01	0.84	0.02	0.94	0.01
		IRTree	0.93	0.01	0.90	0.02	0.95	0.01	0.85	0.02	0.97	0.01
0.5	GPCM	GPCM	0.96	0.01	0.96	0.01	0.96	0.01	0.96	0.01	0.96	0.01
		IRTree	0.96	0.01	0.96	0.01	0.96	0.01	0.96	0.01	0.96	0.01
	IRTree	GPCM	0.93	0.01	0.93	0.01	0.93	0.01	0.93	0.01	0.93	0.01
		IRTree	0.93	0.01	0.93	0.01	0.93	0.01	0.93	0.01	0.93	0.01
	IRTree:Non-Invar.	GPCM	0.93	0.01	0.93	0.01	0.93	0.01	0.93	0.01	0.93	0.01
		IRTree	0.93	0.01	0.93	0.01	0.93	0.01	0.93	0.01	0.93	0.01
0.6	GPCM	GPCM	0.95	0.01	0.96	0.01	0.95	0.01	0.97	0.01	0.94	0.01
		IRTree	0.95	0.01	0.96	0.01	0.95	0.01	0.96	0.01	0.94	0.01
	IRTree	GPCM	0.92	0.01	0.94	0.01	0.90	0.02	0.93	0.01	0.91	0.02
		IRTree	0.93	0.01	0.94	0.01	0.92	0.01	0.95	0.01	0.91	0.02
	IRTree:Non-Invar.	GPCM	0.93	0.01	0.94	0.01	0.90	0.02	0.94	0.01	0.91	0.01
		IRTree	0.93	0.01	0.94	0.01	0.92	0.02	0.95	0.01	0.91	0.01

0.7	GPCM	GPCM	0.95	0.01	0.96	0.01	0.93	0.02	0.97	0.01	0.91	0.02
		IRTree	0.95	0.01	0.96	0.01	0.93	0.02	0.97	0.01	0.91	0.02
	IRTree	GPCM	0.91	0.01	0.95	0.01	0.84	0.03	0.93	0.01	0.87	0.02
		IRTree	0.93	0.01	0.94	0.01	0.91	0.02	0.96	0.01	0.87	0.02
	IRTree:Non-Invar.	GPCM	0.92	0.01	0.95	0.01	0.85	0.03	0.94	0.01	0.88	0.02
		IRTree	0.93	0.01	0.94	0.01	0.91	0.02	0.96	0.01	0.87	0.02
0.8	GPCM	GPCM	0.95	0.01	0.97	0.01	0.90	0.02	0.98	0.01	0.87	0.02
		IRTree	0.95	0.01	0.97	0.01	0.90	0.02	0.98	0.01	0.87	0.02
	IRTree	GPCM	0.91	0.01	0.95	0.01	0.75	0.03	0.94	0.01	0.79	0.03
		IRTree	0.94	0.01	0.95	0.01	0.88	0.02	0.97	0.01	0.82	0.02
	IRTree:Non-Invar.	GPCM	0.91	0.01	0.95	0.01	0.76	0.03	0.94	0.01	0.80	0.03
		IRTree	0.94	0.01	0.95	0.01	0.88	0.02	0.97	0.01	0.82	0.02
0.9	GPCM	GPCM	0.96	0.01	0.98	0.00	0.84	0.04	0.98	0.00	0.81	0.03
		IRTree	0.96	0.01	0.98	0.00	0.84	0.04	0.98	0.00	0.80	0.03
	IRTree	GPCM	0.93	0.01	0.96	0.01	0.64	0.04	0.96	0.00	0.66	0.05
		IRTree	0.95	0.01	0.97	0.00	0.80	0.04	0.98	0.00	0.72	0.03
	IRTree:Non-Invar.	GPCM	0.93	0.01	0.96	0.01	0.63	0.04	0.96	0.00	0.65	0.04
		IRTree	0.95	0.01	0.97	0.00	0.79	0.04	0.98	0.00	0.72	0.03

Table S4. Simulation Results, N = 1000, n = 50.

**Sel. Rate**	**Gen. Mod.**	**Fit Mod.**	**Correct Decision Rate**		**Sensitivity**		**Specificity**		**Positive Predictive Value**		**Negative Predictive Value**	
			**M**	**SD**	**M**	**SD**	**M**	**SD**	**M**	**SD**	**M**	**SD**
0.1	GPCM	GPCM	0.98	0.00	0.91	0.03	0.99	0.00	0.89	0.04	0.99	0.00
		IRTree	0.98	0.00	0.91	0.04	0.99	0.00	0.88	0.03	0.99	0.00
	IRTree	GPCM	0.94	0.01	0.66	0.05	0.97	0.01	0.70	0.04	0.96	0.00
		IRTree	0.96	0.01	0.85	0.03	0.98	0.01	0.80	0.04	0.98	0.00
	IRTree:Non-Invar.	GPCM	0.94	0.01	0.70	0.05	0.97	0.01	0.72	0.05	0.97	0.01
		IRTree	0.96	0.01	0.85	0.04	0.98	0.00	0.81	0.03	0.98	0.00
0.15	GPCM	GPCM	0.98	0.00	0.93	0.02	0.98	0.01	0.91	0.03	0.99	0.00
		IRTree	0.98	0.00	0.93	0.02	0.98	0.01	0.91	0.03	0.99	0.00
	IRTree	GPCM	0.93	0.01	0.71	0.03	0.96	0.01	0.78	0.04	0.95	0.01
		IRTree	0.96	0.01	0.89	0.03	0.97	0.01	0.84	0.03	0.98	0.00
	IRTree:Non-Invar.	GPCM	0.93	0.01	0.74	0.03	0.97	0.01	0.79	0.03	0.96	0.01
		IRTree	0.96	0.01	0.88	0.03	0.97	0.01	0.84	0.03	0.98	0.00
0.2	GPCM	GPCM	0.98	0.01	0.94	0.02	0.98	0.01	0.94	0.02	0.99	0.00
		IRTree	0.98	0.01	0.94	0.02	0.98	0.01	0.94	0.02	0.98	0.00
	IRTree	GPCM	0.92	0.01	0.76	0.03	0.96	0.01	0.83	0.03	0.94	0.01
		IRTree	0.95	0.01	0.91	0.02	0.97	0.01	0.87	0.02	0.98	0.01

	IRTree:Non-Invar.	GPCM	0.93	0.01	0.79	0.03	0.96	0.01	0.85	0.02	0.95	0.01
		IRTree	0.95	0.01	0.91	0.02	0.97	0.01	0.87	0.02	0.98	0.01
0.25	GPCM	GPCM	0.97	0.01	0.95	0.02	0.98	0.01	0.94	0.02	0.98	0.01
		IRTree	0.97	0.01	0.95	0.02	0.98	0.01	0.94	0.02	0.98	0.01
	IRTree	GPCM	0.92	0.01	0.82	0.03	0.96	0.01	0.87	0.02	0.94	0.01
		IRTree	0.95	0.01	0.93	0.02	0.96	0.01	0.89	0.02	0.98	0.01
	IRTree:Non-Invar.	GPCM	0.93	0.01	0.83	0.03	0.96	0.01	0.87	0.02	0.94	0.01
		IRTree	0.95	0.01	0.92	0.02	0.96	0.01	0.89	0.02	0.97	0.01
0.5	GPCM	GPCM	0.97	0.00	0.97	0.01	0.97	0.01	0.97	0.01	0.97	0.01
		IRTree	0.97	0.01	0.97	0.01	0.97	0.01	0.97	0.01	0.97	0.01
	IRTree	GPCM	0.95	0.01	0.95	0.01	0.95	0.01	0.95	0.01	0.95	0.01
		IRTree	0.95	0.01	0.95	0.01	0.95	0.01	0.95	0.01	0.95	0.01
	IRTree:Non-Invar.	GPCM	0.95	0.01	0.95	0.01	0.95	0.01	0.95	0.01	0.95	0.01
		IRTree	0.95	0.01	0.95	0.01	0.95	0.01	0.95	0.01	0.95	0.01
0.6	GPCM	GPCM	0.97	0.01	0.97	0.01	0.96	0.01	0.97	0.01	0.96	0.01
		IRTree	0.97	0.01	0.97	0.01	0.96	0.01	0.97	0.01	0.95	0.01
	IRTree	GPCM	0.94	0.01	0.96	0.01	0.92	0.02	0.95	0.01	0.94	0.01
		IRTree	0.95	0.01	0.95	0.01	0.94	0.02	0.96	0.01	0.93	0.01
	IRTree:Non-Invar.	GPCM	0.94	0.01	0.96	0.01	0.92	0.02	0.95	0.01	0.94	0.01
		IRTree	0.95	0.01	0.95	0.01	0.94	0.01	0.96	0.01	0.93	0.01
0.7	GPCM	GPCM	0.97	0.01	0.97	0.01	0.95	0.01	0.98	0.01	0.94	0.02
		IRTree	0.97	0.01	0.97	0.01	0.95	0.01	0.98	0.01	0.94	0.02
	IRTree	GPCM	0.93	0.01	0.96	0.01	0.85	0.02	0.94	0.01	0.90	0.02
		IRTree	0.95	0.01	0.96	0.01	0.93	0.02	0.97	0.01	0.91	0.02
	IRTree:Non-Invar.	GPCM	0.93	0.01	0.96	0.01	0.87	0.02	0.94	0.01	0.91	0.02
		IRTree	0.95	0.01	0.96	0.01	0.93	0.01	0.97	0.01	0.91	0.02
0.8	GPCM	GPCM	0.97	0.01	0.98	0.01	0.93	0.02	0.98	0.01	0.91	0.02
		IRTree	0.97	0.01	0.97	0.01	0.93	0.02	0.98	0.01	0.90	0.02
	IRTree	GPCM	0.92	0.01	0.96	0.01	0.76	0.03	0.94	0.01	0.83	0.02
		IRTree	0.95	0.01	0.97	0.01	0.90	0.02	0.98	0.01	0.87	0.02
	IRTree:Non-Invar.	GPCM	0.92	0.01	0.96	0.01	0.76	0.03	0.94	0.01	0.83	0.03
		IRTree	0.95	0.01	0.97	0.01	0.91	0.02	0.98	0.01	0.87	0.02
0.9	GPCM	GPCM	0.97	0.01	0.98	0.00	0.88	0.04	0.99	0.00	0.86	0.03
		IRTree	0.97	0.01	0.98	0.00	0.88	0.04	0.99	0.00	0.85	0.03
	IRTree	GPCM	0.93	0.01	0.97	0.01	0.65	0.04	0.96	0.00	0.69	0.04
		IRTree	0.96	0.01	0.98	0.01	0.85	0.04	0.98	0.00	0.80	0.04
	IRTree:Non-Invar.	GPCM	0.94	0.01	0.97	0.01	0.66	0.04	0.96	0.00	0.69	0.04
		IRTree	0.96	0.01	0.98	0.01	0.85	0.04	0.98	0.00	0.79	0.04

Table S5. Simulation Results, N = 2000, n = 25.

Sel. Rate	Gen. Mod.	Fit Mod.	Correct Decision Rate		Sensitivity		Specificity		Positive Predictive Value		Negative Predictive Value	
			**M**	**SD**	**M**	**SD**	**M**	**SD**	**M**	**SD**	**M**	**SD**
0.1	GPCM	GPCM	0.97	0.00	0.88	0.03	0.98	0.00	0.85	0.03	0.99	0.00
		IRTree	0.97	0.00	0.88	0.03	0.98	0.00	0.84	0.03	0.99	0.00
	IRTree	GPCM	0.93	0.01	0.63	0.03	0.96	0.00	0.65	0.03	0.96	0.00
		IRTree	0.95	0.00	0.80	0.03	0.97	0.00	0.72	0.02	0.98	0.00
	IRTree:Non-Invar.	GPCM	0.93	0.01	0.67	0.03	0.96	0.00	0.67	0.03	0.96	0.00
		IRTree	0.95	0.00	0.80	0.03	0.97	0.00	0.72	0.02	0.98	0.00
0.15	GPCM	GPCM	0.97	0.00	0.92	0.02	0.98	0.00	0.88	0.02	0.99	0.00
		IRTree	0.97	0.00	0.91	0.02	0.98	0.00	0.88	0.02	0.98	0.00
	IRTree	GPCM	0.92	0.01	0.70	0.03	0.96	0.01	0.74	0.03	0.95	0.00
		IRTree	0.94	0.01	0.85	0.02	0.96	0.00	0.78	0.02	0.97	0.00
	IRTree:Non-Invar.	GPCM	0.92	0.01	0.72	0.02	0.96	0.00	0.74	0.02	0.95	0.00
		IRTree	0.94	0.01	0.85	0.02	0.96	0.01	0.78	0.02	0.97	0.00
0.2	GPCM	GPCM	0.96	0.00	0.93	0.02	0.97	0.00	0.90	0.02	0.98	0.00
		IRTree	0.96	0.00	0.92	0.02	0.97	0.00	0.90	0.02	0.98	0.00
	IRTree	GPCM	0.91	0.01	0.75	0.03	0.95	0.01	0.80	0.02	0.94	0.01
		IRTree	0.94	0.01	0.88	0.02	0.95	0.00	0.82	0.02	0.97	0.00
	IRTree:Non-Invar.	GPCM	0.92	0.01	0.78	0.02	0.95	0.01	0.80	0.02	0.94	0.01
		IRTree	0.94	0.01	0.88	0.02	0.95	0.01	0.81	0.02	0.97	0.00
0.25	GPCM	GPCM	0.96	0.00	0.94	0.01	0.97	0.01	0.92	0.01	0.98	0.00
		IRTree	0.96	0.00	0.93	0.01	0.97	0.01	0.92	0.01	0.98	0.00
	IRTree	GPCM	0.91	0.01	0.80	0.02	0.95	0.01	0.84	0.02	0.93	0.01
		IRTree	0.93	0.01	0.90	0.01	0.95	0.01	0.85	0.02	0.97	0.00
	IRTree:Non-Invar.	GPCM	0.92	0.01	0.81	0.02	0.95	0.01	0.84	0.02	0.94	0.01
		IRTree	0.93	0.01	0.90	0.02	0.95	0.01	0.85	0.02	0.96	0.01
0.5	GPCM	GPCM	0.96	0.01	0.95	0.01	0.96	0.01	0.96	0.01	0.95	0.01
		IRTree	0.96	0.01	0.96	0.01	0.96	0.01	0.96	0.01	0.96	0.01
	IRTree	GPCM	0.93	0.01	0.93	0.01	0.93	0.01	0.93	0.01	0.93	0.01
		IRTree	0.93	0.01	0.93	0.01	0.93	0.01	0.93	0.01	0.93	0.01
	IRTree:Non-Invar.	GPCM	0.93	0.01	0.93	0.01	0.93	0.01	0.93	0.01	0.93	0.01
		IRTree	0.93	0.01	0.93	0.01	0.93	0.01	0.93	0.01	0.93	0.01
0.6	GPCM	GPCM	0.95	0.01	0.96	0.01	0.95	0.01	0.97	0.01	0.94	0.01
		IRTree	0.95	0.01	0.96	0.01	0.95	0.01	0.96	0.01	0.94	0.01
	IRTree	GPCM	0.93	0.01	0.94	0.01	0.90	0.01	0.94	0.01	0.91	0.01
		IRTree	0.93	0.01	0.94	0.01	0.92	0.01	0.95	0.01	0.91	0.01
	IRTree:Non-Invar.	GPCM	0.93	0.01	0.94	0.01	0.91	0.01	0.94	0.01	0.91	0.01
		IRTree	0.93	0.01	0.94	0.01	0.92	0.01	0.95	0.01	0.91	0.01

	GPCM	GPCM	0.95	0.01	0.96	0.01	0.93	0.01	0.97	0.01	0.91	0.01
		IRTree	0.95	0.01	0.96	0.01	0.93	0.01	0.97	0.01	0.91	0.01
0.7	IRTree	GPCM	0.92	0.01	0.95	0.01	0.84	0.02	0.93	0.01	0.87	0.02
		IRTree	0.93	0.01	0.94	0.01	0.91	0.01	0.96	0.00	0.88	0.01
	IRTree:Non-Invar.	GPCM	0.92	0.01	0.95	0.01	0.85	0.02	0.94	0.01	0.87	0.01
		IRTree	0.93	0.01	0.94	0.01	0.91	0.01	0.96	0.01	0.87	0.01
	GPCM	GPCM	0.95	0.01	0.97	0.01	0.91	0.02	0.98	0.00	0.87	0.02
		IRTree	0.95	0.01	0.97	0.00	0.90	0.02	0.98	0.00	0.87	0.02
0.8	IRTree	GPCM	0.91	0.01	0.95	0.01	0.74	0.03	0.94	0.01	0.79	0.02
		IRTree	0.94	0.01	0.95	0.01	0.88	0.02	0.97	0.00	0.82	0.02
	IRTree:Non-Invar.	GPCM	0.91	0.01	0.95	0.01	0.75	0.03	0.94	0.01	0.80	0.02
		IRTree	0.94	0.01	0.95	0.00	0.88	0.02	0.97	0.00	0.82	0.02
	GPCM	GPCM	0.96	0.00	0.98	0.00	0.84	0.03	0.98	0.00	0.80	0.02
		IRTree	0.96	0.00	0.98	0.00	0.84	0.03	0.98	0.00	0.80	0.02
0.9	IRTree	GPCM	0.93	0.01	0.96	0.00	0.64	0.04	0.96	0.00	0.65	0.04
		IRTree	0.95	0.00	0.97	0.00	0.80	0.03	0.98	0.00	0.72	0.03
	IRTree:Non-Invar.	GPCM	0.93	0.01	0.96	0.00	0.63	0.03	0.96	0.00	0.65	0.03
		IRTree	0.95	0.01	0.97	0.00	0.80	0.03	0.98	0.00	0.72	0.02

Table S6. Simulation Results, N = 2000, n = 50.

**Sel. Rat e**	**Gen. Mod.**	**Fit Mod.**	**Correct Decision Rate**		**Sensitivity**		**Specificity**		**Positive Predictive Value**		**Negative Predictive Value**	
			**M**	**SD**	**M**	**SD**	**M**	**SD**	**M**	**SD**	**M**	**SD**
	GPCM	GPCM	0.98	0.00	0.92	0.02	0.99	0.00	0.89	0.02	0.99	0.00
		IRTree	0.98	0.00	0.92	0.02	0.99	0.00	0.88	0.02	0.99	0.00
0.1	IRTree	GPCM	0.94	0.01	0.65	0.03	0.97	0.00	0.70	0.03	0.96	0.00
		IRTree	0.96	0.00	0.85	0.03	0.98	0.00	0.80	0.02	0.98	0.00
	IRTree:Non-Invar.	GPCM	0.94	0.01	0.69	0.03	0.97	0.00	0.72	0.03	0.97	0.00
		IRTree	0.96	0.00	0.85	0.03	0.98	0.00	0.80	0.03	0.98	0.00
	GPCM	GPCM	0.98	0.00	0.93	0.02	0.98	0.00	0.92	0.02	0.99	0.00
		IRTree	0.98	0.00	0.93	0.02	0.98	0.00	0.91	0.02	0.99	0.00
0.15	IRTree	GPCM	0.93	0.01	0.71	0.03	0.96	0.00	0.77	0.03	0.95	0.00
		IRTree	0.96	0.00	0.89	0.02	0.97	0.00	0.84	0.02	0.98	0.00
	IRTree:Non-Invar.	GPCM	0.93	0.01	0.75	0.02	0.97	0.00	0.79	0.02	0.96	0.00
		IRTree	0.96	0.00	0.89	0.02	0.97	0.00	0.84	0.02	0.98	0.00
	GPCM	GPCM	0.97	0.00	0.95	0.01	0.98	0.00	0.93	0.02	0.99	0.00
0.2		IRTree	0.98	0.00	0.94	0.01	0.98	0.00	0.93	0.01	0.99	0.00
	IRTree	GPCM	0.92	0.01	0.76	0.02	0.96	0.01	0.83	0.02	0.94	0.01
		IRTree	0.96	0.01	0.91	0.02	0.97	0.00	0.87	0.02	0.98	0.00

	IRTree:Non-Invar.	GPCM	0.93	0.01	0.79	0.02	0.96	0.00	0.84	0.02	0.95	0.01
		IRTree	0.96	0.00	0.91	0.01	0.97	0.00	0.87	0.02	0.98	0.00
0.25	GPCM	GPCM	0.97	0.00	0.95	0.01	0.98	0.01	0.94	0.01	0.98	0.00
		IRTree	0.97	0.00	0.95	0.01	0.98	0.00	0.95	0.01	0.98	0.00
	IRTree	GPCM	0.92	0.01	0.81	0.02	0.96	0.01	0.87	0.02	0.94	0.01
		IRTree	0.95	0.00	0.92	0.01	0.96	0.01	0.89	0.01	0.97	0.00
	IRTree:Non-Invar.	GPCM	0.93	0.01	0.83	0.02	0.96	0.01	0.88	0.02	0.94	0.01
		IRTree	0.95	0.01	0.93	0.01	0.96	0.01	0.89	0.01	0.98	0.00
0.5	GPCM	GPCM	0.97	0.00	0.97	0.01	0.97	0.01	0.97	0.01	0.97	0.01
		IRTree	0.97	0.00	0.97	0.01	0.97	0.01	0.97	0.01	0.97	0.01
	IRTree	GPCM	0.95	0.00	0.95	0.01	0.95	0.01	0.95	0.01	0.95	0.01
		IRTree	0.95	0.00	0.95	0.01	0.95	0.01	0.95	0.01	0.95	0.01
	IRTree:Non-Invar.	GPCM	0.95	0.01	0.95	0.01	0.95	0.01	0.95	0.01	0.95	0.01
		IRTree	0.95	0.01	0.95	0.01	0.95	0.01	0.95	0.01	0.95	0.01
0.6	GPCM	GPCM	0.97	0.00	0.97	0.01	0.96	0.01	0.97	0.01	0.96	0.01
		IRTree	0.97	0.00	0.97	0.01	0.96	0.01	0.97	0.01	0.96	0.01
	IRTree	GPCM	0.94	0.01	0.96	0.01	0.92	0.01	0.95	0.01	0.94	0.01
		IRTree	0.95	0.00	0.96	0.01	0.95	0.01	0.96	0.01	0.93	0.01
	IRTree:Non-Invar.	GPCM	0.94	0.01	0.96	0.01	0.92	0.01	0.95	0.01	0.94	0.01
		IRTree	0.95	0.01	0.96	0.01	0.94	0.01	0.96	0.01	0.93	0.01
0.7	GPCM	GPCM	0.97	0.00	0.97	0.01	0.95	0.01	0.98	0.00	0.94	0.01
		IRTree	0.97	0.00	0.97	0.01	0.95	0.01	0.98	0.00	0.94	0.01
	IRTree	GPCM	0.93	0.01	0.96	0.01	0.86	0.02	0.94	0.01	0.90	0.02
		IRTree	0.95	0.01	0.96	0.01	0.94	0.01	0.97	0.00	0.91	0.01
	IRTree:Non-Invar.	GPCM	0.93	0.01	0.96	0.01	0.87	0.02	0.94	0.01	0.91	0.01
		IRTree	0.95	0.00	0.96	0.01	0.94	0.01	0.97	0.00	0.91	0.01
0.8	GPCM	GPCM	0.97	0.00	0.98	0.00	0.93	0.02	0.98	0.00	0.91	0.02
		IRTree	0.97	0.00	0.98	0.00	0.93	0.02	0.98	0.00	0.91	0.02
	IRTree	GPCM	0.92	0.01	0.96	0.01	0.76	0.02	0.94	0.01	0.83	0.02
		IRTree	0.96	0.00	0.97	0.01	0.91	0.02	0.98	0.00	0.87	0.02
	IRTree:Non-Invar.	GPCM	0.92	0.01	0.96	0.00	0.76	0.02	0.94	0.01	0.84	0.02
		IRTree	0.96	0.00	0.97	0.00	0.91	0.01	0.98	0.00	0.87	0.02
0.9	GPCM	GPCM	0.97	0.00	0.98	0.00	0.89	0.03	0.99	0.00	0.86	0.02
		IRTree	0.97	0.00	0.98	0.00	0.89	0.02	0.99	0.00	0.86	0.02
	IRTree	GPCM	0.94	0.01	0.97	0.00	0.65	0.03	0.96	0.00	0.69	0.04
		IRTree	0.96	0.00	0.98	0.00	0.85	0.03	0.98	0.00	0.80	0.02
	IRTree:Non-Invar.	GPCM	0.94	0.01	0.97	0.00	0.66	0.03	0.96	0.00	0.70	0.03
		IRTree	0.96	0.00	0.98	0.00	0.85	0.03	0.98	0.00	0.80	0.02
